# Supplementary material for: Three-Dimensional Stress Fields in Thick Orthotropic Plates with Sharply Curved Notches under In-Plane and Out-of-Plane Shear
Source: Polymers (Basel). 2023 Apr 24;15(9):2013. doi: 10.3390/polym15092013 (PMC10181459; doi:10.3390/polym15092013)
Supplement: Supplementary file 1 [file polymers-15-02013-s001.zip › polymers-2239788-supplementary.pdf]

## S1. Generation of the FE models

In the following sections the ANSYS APDL scripts needed for generating the FE model of a disc with semi-elliptical notch are reported, alongside a graphical explanation of the input parameters. After that, the information concerning the applied Boundary Conditions and the detailed information about each FE model are reported. Finally, the information about hyperbolic notches are presented.

### S1.1. APDL for generating a semi-elliptical notch in 2D

```
/PREP7

!**********************************************************
/COM, data
pacmanOX = 5                ![mm] X-coordinate for disk center
pacmanR  = 5                ![mm] radius of the disk
roh = 1                     ![mm] notch tip radius
geom = 'ell1'
notchW = 5                  ![mm] half-width of the notch ellipse
innerMeshRadius = 1         ![mm] radius of the inner circle
keepHalfPacman = 0          ! 1:yes, 0:no

notchH = (roh*notchW)**0.5 ![mm] half-heigth of the notch

coronaDiv         = 40
externalRadDiv    = 50
externalRadSR     = 5
innerRadDiv       = 100
innerRadSR        = 10
!**********************************************************

/COM, create lines
K,,pacmanOX,0,0
CIRCLE,1,innerMeshRadius

/COM, create areas
CYL4,pacmanOX,0,pacmanR
CYL4,0,0,notchH
ARSCALE,2, , ,notchW/notchH,1,1, ,0,1

/COM, divide pacman center with inner Radius
LSEL, S,,,1,4,1
ASBL, 1, ALL

/COM, remove notch-area from pacman
AOVLAP,ALL
ADELE,1
ADELE,6,7,1

/COM, segment areas
```

```
L,10,2
L,3,7
L,2,6
L,5,9
LSEL, S,,,2,6
LSEL, A,,,5,8
ASBL, ALL, ALL

/COM,delete lower half of Pacman, if requested
*IF, keepHalfPacman, EQ, 0, THEN
ADELE,6,7
ADELE,3

!*********************************************************
/COM, perform mesh

LSEL, S,,,5
LSEL, A,,,1
LESIZE,ALL, , ,coronaDiv,1, , , ,1

LSEL, S,,,25
LSEL, A,,,17
LESIZE,ALL, , ,coronaDiv/2,1, , , ,1

LSEL, S,,,3
LSEL, A,,,6
LSEL, A,,,23
LESIZE,ALL, , ,externalRadDiv,externalRadSR, , , ,1

LSEL, S,,,2
LSEL, A,,,15
LESIZE,ALL, , ,innerRadDiv,innerRadSR, , , ,1

*ENDIF

/COM, create element
ET,1,PLANE183

AMESH, ALL

/COM, save model info
*GET, E_COUNT, ELEM,,COUNT
*GET, N_COUNT, NODE,,COUNT
```

**S1.2. Description of the input parameters for the 2D APDL**

In Figures S1 and S2 a graphical representation of the APDL script input is shown, while Table S1 is complementary to Figure S2.

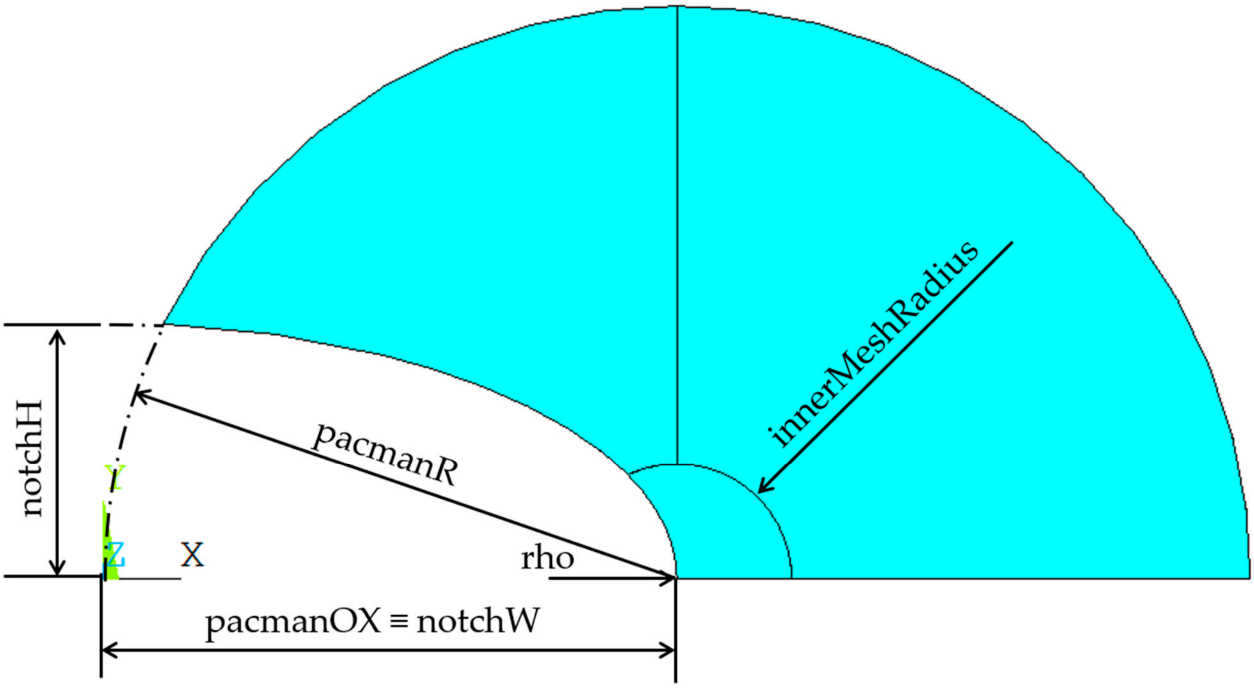


**Figure S1.** Graphical representation of the input for the APDL script.

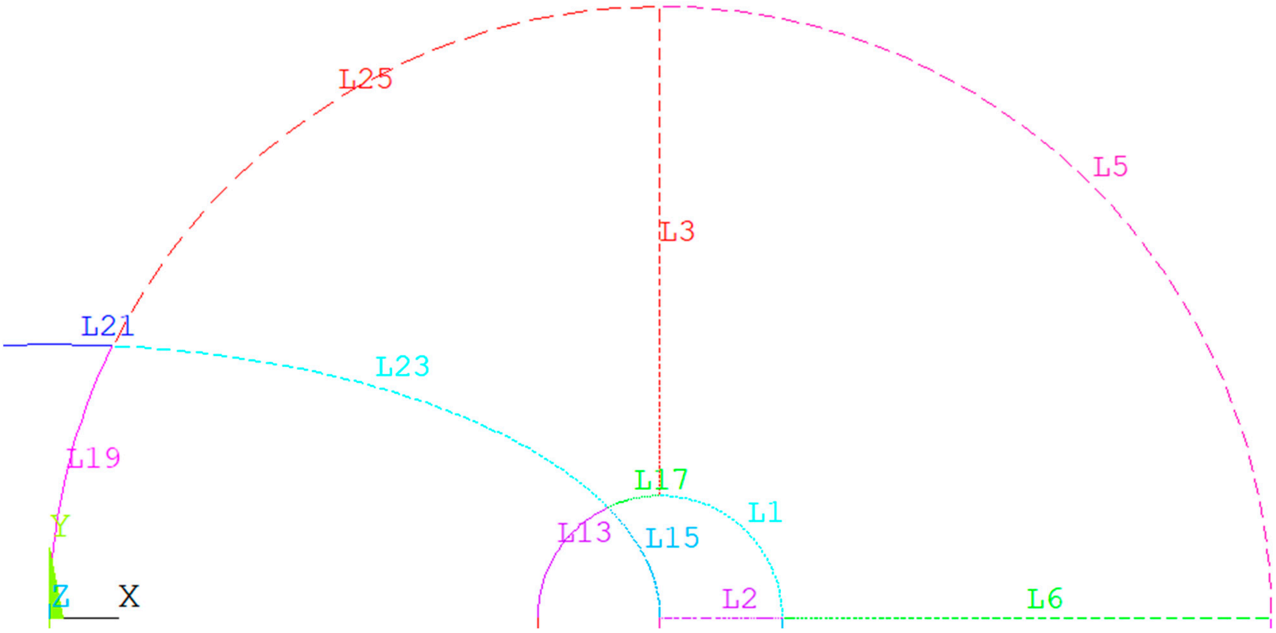


**Figure S2.** Graphical representation of the input for the APDL script.

**Table S1.** Properties of the materials used in the numerical analyses.

| Line | Number of elements | Spacing ratio |
| --- | --- | --- |
| 5 | coronaDiv | 1 |
| 1 | coronaDiv | 1 |
| 25 | coronaDiv/2 | 1 |
| 17 | coronaDiv/2 | 1 |
| 3 | externalRadDiv | externalRadSR |
| 6 | externalRadDiv | externalRadSR |
| 23 | externalRadDiv | externalRadSR |
| 2 | innerRadDiv | innerRadSR |
| 15 | innerRadDiv | innerRadSR |

**S1.3. APDL for extruding the model to a 3D thick plate**

```
/COM, set options
ettt = 30        !Number of elements through-the-thickness
sprat = 60       !Spacing ratio
plthick = 1      ![mm] Plate thickness: it will be divided by 2

/COM, set element type
ET,2,SOLID186

/COM, set extrude options
TYPE,   2   !Element type 2
EXTOPT,ESIZE,ettt,sprat,
EXTOPT,ACLEAR,1    !Delete area upon extrusion

EXTOPT,ATTR,0,0,0
MAT,1
REAL,_Z4
ESYS,0

/COM, Extrude volumes
ASEL,ALL
VEXT,ALL, , ,0,0,plthick/2,,,,

/COM, save model info
*GET, E_COUNT, ELEM,,COUNT
*GET, N_COUNT, NODE,,COUNT
```

### S1.4. Output of the APDL scripts

In Figure S3 the model obtained from running the two APDL scripts is represented. Relevant properties for through-the-thickness meshing are shown in the first lines of the previous section, alongside comments for describing their meaning. For the 2D model ANSYS free-mesher was used, with the properties reported into the script. Eventually, it resulted in a mapped mesh for the elements at a distance greater than innerMeshRadius from the disc center.

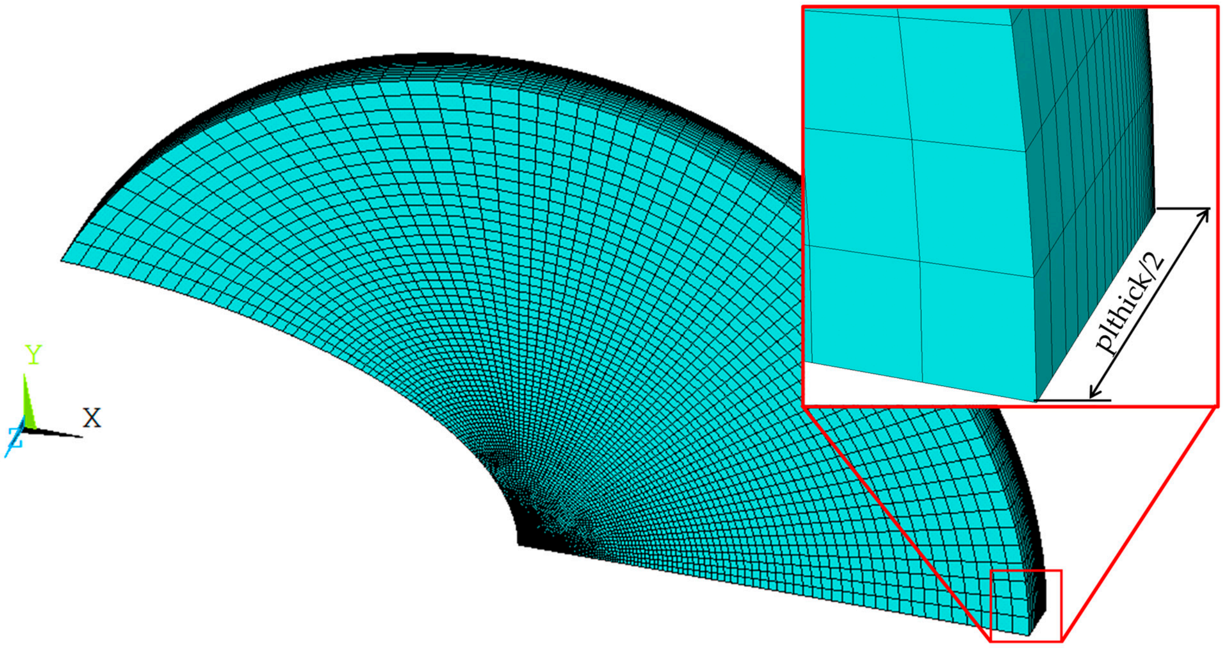


**Figure S3.** Graphical representation of the complete FE model of the semi-elliptical notch, generated with the scripts reported above.

### S1.5. Application of the Boundary Conditions

The loads were applied as nodal displacements on the circular boundary of the disk. In more detail, nodal coordinates of the boundary were collected in ANSYS APDL and, by means of an external script, the displacements to be applied were evaluated according to the expressions reported in the Appendix A of the paper. Eventually, another APDL script was used to apply the nodal displacements.

For simulating Mode 2, the symmetry of the model was exploited. Symmetry boundary conditions were applied to the XY face of the model, at Z-coordinate equal to plthick/2. In fact, within this Supplementary Material Z=0 identifies a disk external surface. Anti-symmetry was applied to the nodes lying on the plane XZ.

For simulating Mode 3, only the anti-symmetry of plane XZ was exploited. Hence, the model was mirrored about that face and doubled its size.

### S1.6. Modelling of hyperbolic notches

Differently from the case of elliptical notches, no direct modelling of hyperbolic curves is available in ANSYS APDL. To overcome this limitation, each curve was discretized in several points that were later connected with a spline curve. For the rest, the procedure is the same as that previously presented for semi-elliptical notches. The list of keypoints used for each hyperbolic curve is reported in the Appendix S1-A in Tables S1-A1, S1-A2, S1-A3.

For convenience, the center of the disk is placed at a distance $r_0$ from the notch tip, as per Figure 2(c).

### S1.7. Modelling parameters for each notched geometry

The parameters used for modelling each geometry are reported in Tables S2 and S3.

**S1.8. Post processing ADPL**

To provide a full description of the information retrieved from the FE model, the following script reports the operations executed on the model after running the simulation.

```
/POST1

saveName = STRCAT(geom,'_')
saveName = STRCAT(saveName,matId)
saveName = STRCAT(saveName,'_')
saveName = STRCAT(saveName,spost)
saveName = STRCAT(saveName,'_')
saveName = STRCAT(saveName,'t')
saveName = STRCAT(saveName,CHRVAL(plthick))
saveName = STRCAT(saveName,'_')
saveName = STRCAT(saveName,'e')
saveName = STRCAT(saveName,CHRVAL(ettt))
saveName = STRCAT(saveName,'_')
saveName = STRCAT(saveName,'sp')
saveName = STRCAT(saveName,CHRVAL(sprat))


/SYP,MKDIR,saveName
/INQUIRE, curDir, DIRECTORY --
stringDir = curDir(1)
/CWD, JOIN(stringDir,saveName)

/COM, Load results
!SET, FIRST          !Its use depends on the Ansys version

/COM, Save
SAVE,saveName,db,,ALL

/OUTPUT, STRCAT(saveName,'_MPLIST'),txt,,
MPLIST,ALL
/OUTPUT

/COM, update overall node number for following loop
ALLSEL,ALL
*GET, nodeNum,NODE,,COUNT

/COM, Extract nodes from lower surface
ASEL,S,,,8
ASEL,A,,,12
ASEL,A,,,7
ASEL,A,,,15
NSLA,S,1
```

```
/COM, first option generates the usual node list, while the second option creates a list with higher coordinate precision
*IF 1,EQ,0,THEN
        /OUTPUT, STRCAT(saveName,'_NLIST'),lis,,
        NLIST,ALL
        /OUTPUT
*ELSE
*GET, selNod, NSEL  !get the number of selected nodes
*DIM, nodeTable,TABLE,selNod,4  !create a table only for the selected nodes

NCounter = 0
*DO,i,1,nodeNum
*GET,NisSelected,NODE,i,NSEL,,
*IF,NisSelected,GE,0.99,THEN
                nodeTable(NCounter,1)=i
                *GET,nodeTable(NCounter,2),NODE,i,LOC,X
                *GET,nodeTable(NCounter,3),NODE,i,LOC,Y
                *GET,nodeTable(NCounter,4),NODE,i,LOC,Z
                NCounter = NCounter + 1
*ENDIF
*ENDDO

*MWRITE,nodeTable,STRCAT(saveName,'_NLIST'),lis,,,4,
%I      %f      %f      %f

*ENDIF


/COM, Extract stress components for the nodes
/OUTPUT, STRCAT(saveName,'_PRNSOL'),lis,,
PRNSOL,S,ALL
/OUTPUT

/COM, Extract min SYZ along notch tip
LSEL,S,,,32
NSLL,S,1
/OUTPUT, STRCAT(saveName,'_tip'),txt,,
PRNSOL,S,ALL
/OUTPUT

/SHOW,PNG
EPLOT

/EXIT
```

**Table S2.** Parameters of the FE models for semi-elliptical notches. notchW = 5mm.

| rho [mm] | material | coronaDiv | externalRadDiv | externalRadSR | innerRadDiv | innerRadSR | plthick [mm] | ettt | sprat |
|---|---|---|---|---|---|---|---|---|---|
| 1 | 1 | 40 | 50 | 5 | 100 | 10 | 1 | 20 | 40 |
| 0.1 | 1 | 25 | 30 | 5 | 100 | 90 | 1 | 20 | 40 |
| 0.01 | 1 | 25 | 23 | 5 | 100 | 90 | 1 | 20 | 20 |
| 0.01 | 1 | 25 | 23 | 5 | 100 | 90 | 5 | 20 | 20 |
| 0.01 | 1 | 25 | 23 | 5 | 100 | 90 | 10 | 25 | 20 |
| 0.01 | 2 | 25 | 23 | 5 | 100 | 90 | 1 | 40 | 60 |
| 0.01 | 2 | 25 | 23 | 5 | 100 | 90 | 5 | 40 | 80 |
| 0.01 | 2 | 25 | 23 | 5 | 100 | 90 | 10 | 50 | 80 |
| 0.001 | 1 | 25 | 23 | 5 | 100 | 90 | 1 | 40 | 60 |
| 0.001 | 2 | 25 | 23 | 5 | 100 | 90 | 1 | 50 | 80 |
| 0.001 | 3 | 25 | 23 | 5 | 100 | 90 | 1 | 40 | 60 |

**Table S3.** Parameters of the FE models for hyperbolic notches. notchW = 10mm.

| $2\alpha$[deg] | material | coronaDiv | exter-nalRadDiv | exter-nalRadSR | innerRadDiv | innerRadSR | plthick [mm] | ettt | sprat |
|---|---|---|---|---|---|---|---|---|---|
| 45 | 1 | 25 | 23 | 5 | 100 | 90 | 1 | 25 | 80 |
| 90 | 2 | 25 | 23 | 5 | 100 | 90 | 1 | 30 | 90 |
| 60 | 3 | 25 | 23 | 5 | 100 | 90 | 1 | 30 | 60 |

## Appendix S1-A

**Table S1-A1.** Keypoints used for generating an hyperbolic notch with opening angle $2\alpha$ = 90 degrees and radius of the notch tip equal to 0.01mm.

| X | Y |
|---|---|
| 0.003333 | 0 |
| 0.003333 | 0.00001 |
| 0.003333 | 2E-05 |
| 0.003333 | 3E-05 |
| 0.003333 | 4E-05 |
| 0.003333 | 5E-05 |
| 0.003333 | 6E-05 |
| 0.003333 | 7E-05 |
| 0.003333 | 8E-05 |
| 0.003333 | 9E-05 |
| 0.003333 | 0.0001 |
| 0.003333 | 0.00011 |
| 0.003333 | 0.00012 |
| 0.003332 | 0.00013 |
| 0.003332 | 0.00014 |
| 0.003332 | 0.00015 |
| 0.003332 | 0.00016 |
| 0.003332 | 0.00017 |
| 0.003332 | 0.00018 |
| 0.003332 | 0.00019 |
| 0.003331 | 0.0002 |

| | |
|---|---|
| 0.003331 | 0.00021 |
| 0.003331 | 0.00022 |
| 0.003331 | 0.00023 |
| 0.00333 | 0.00024 |
| 0.00333 | 0.00025 |
| 0.00333 | 0.00026 |
| 0.00333 | 0.00027 |
| 0.003329 | 0.00028 |
| 0.003329 | 0.00029 |
| 0.003329 | 0.0003 |
| 0.003329 | 0.00031 |
| 0.003328 | 0.00032 |
| 0.003328 | 0.000331 |
| 0.003328 | 0.000341 |
| 0.003327 | 0.000351 |
| 0.003327 | 0.000361 |
| 0.003326 | 0.000371 |
| 0.003326 | 0.000381 |
| 0.003326 | 0.000391 |
| 0.003325 | 0.000401 |
| 0.003325 | 0.000411 |
| 0.003324 | 0.000421 |
| 0.003324 | 0.000431 |
| 0.003324 | 0.000441 |
| 0.003323 | 0.000451 |
| 0.003323 | 0.000461 |
| 0.003322 | 0.000472 |
| 0.003322 | 0.000482 |
| 0.003321 | 0.000492 |
| 0.003321 | 0.000502 |
| 0.00332 | 0.000512 |
| 0.00332 | 0.000522 |
| 0.003319 | 0.000532 |
| 0.003319 | 0.000542 |
| 0.003318 | 0.000553 |
| 0.003318 | 0.000563 |
| 0.003317 | 0.000573 |
| 0.003316 | 0.000583 |
| 0.003316 | 0.000593 |
| 0.003315 | 0.000603 |
| 0.003315 | 0.000613 |
| 0.003314 | 0.000624 |
| 0.003313 | 0.000634 |
| 0.003313 | 0.000644 |
| 0.003312 | 0.000654 |
| 0.003311 | 0.000664 |
| 0.003311 | 0.000675 |
| 0.00331 | 0.000685 |
| 0.003309 | 0.000695 |

| | |
|---|---|
| 0.003309 | 0.000705 |
| 0.003308 | 0.000715 |
| 0.003307 | 0.000726 |
| 0.003306 | 0.000736 |
| 0.003306 | 0.000746 |
| 0.003305 | 0.000756 |
| 0.003304 | 0.000767 |
| 0.003303 | 0.000777 |
| 0.003302 | 0.000787 |
| 0.003302 | 0.000797 |
| 0.003301 | 0.000808 |
| 0.0033 | 0.000818 |
| 0.003299 | 0.000828 |
| 0.003298 | 0.000839 |
| 0.003297 | 0.000849 |
| 0.003297 | 0.000859 |
| 0.003296 | 0.00087 |
| 0.003295 | 0.00088 |
| 0.003294 | 0.00089 |
| 0.003293 | 0.000901 |
| 0.003292 | 0.000911 |
| 0.003291 | 0.000921 |
| 0.00329 | 0.000932 |
| 0.003289 | 0.000942 |
| 0.003288 | 0.000953 |
| 0.003287 | 0.000963 |
| 0.003286 | 0.000973 |
| 0.003285 | 0.000984 |
| 0.003284 | 0.000994 |
| 0.003283 | 0.001005 |
| 0.003282 | 0.001015 |
| 0.003281 | 0.001026 |
| 0.00328 | 0.001036 |
| 0.003279 | 0.001047 |
| 0.003278 | 0.001057 |
| 0.003277 | 0.001068 |
| 0.003276 | 0.001078 |
| 0.003274 | 0.001089 |
| 0.003273 | 0.001099 |
| 0.003272 | 0.00111 |
| 0.003271 | 0.00112 |
| 0.00327 | 0.001131 |
| 0.003269 | 0.001142 |
| 0.003267 | 0.001152 |
| 0.003266 | 0.001163 |
| 0.003265 | 0.001173 |
| 0.003264 | 0.001184 |
| 0.003263 | 0.001195 |
| 0.003261 | 0.001205 |

| | |
|---|---|
| 0.00326 | 0.001216 |
| 0.003259 | 0.001227 |
| 0.003257 | 0.001237 |
| 0.003256 | 0.001248 |
| 0.003255 | 0.001259 |
| 0.003254 | 0.001269 |
| 0.003252 | 0.00128 |
| 0.003251 | 0.001291 |
| 0.003249 | 0.001302 |
| 0.003248 | 0.001312 |
| 0.003247 | 0.001323 |
| 0.003245 | 0.001334 |
| 0.003244 | 0.001345 |
| 0.003242 | 0.001356 |
| 0.003241 | 0.001366 |
| 0.00324 | 0.001377 |
| 0.003238 | 0.001388 |
| 0.003237 | 0.001399 |
| 0.003235 | 0.00141 |
| 0.003234 | 0.001421 |
| 0.003232 | 0.001432 |
| 0.003231 | 0.001443 |
| 0.003229 | 0.001454 |
| 0.003227 | 0.001465 |
| 0.003226 | 0.001476 |
| 0.003224 | 0.001487 |
| 0.003223 | 0.001498 |
| 0.003221 | 0.001509 |
| 0.003219 | 0.00152 |
| 0.003218 | 0.001531 |
| 0.003216 | 0.001542 |
| 0.003214 | 0.001553 |
| 0.003213 | 0.001564 |
| 0.003211 | 0.001575 |
| 0.003209 | 0.001586 |
| 0.003208 | 0.001597 |
| 0.003206 | 0.001608 |
| 0.003204 | 0.00162 |
| 0.003202 | 0.001631 |
| 0.003201 | 0.001642 |
| 0.003199 | 0.001653 |
| 0.003197 | 0.001664 |
| 0.003195 | 0.001676 |
| 0.003193 | 0.001687 |
| 0.003191 | 0.001698 |
| 0.00319 | 0.00171 |
| 0.003188 | 0.001721 |
| 0.003186 | 0.001732 |
| 0.003184 | 0.001744 |

| | |
|---|---|
| 0.003182 | 0.001755 |
| 0.00318 | 0.001766 |
| 0.003178 | 0.001778 |
| 0.003176 | 0.001789 |
| 0.003174 | 0.001801 |
| 0.003172 | 0.001812 |
| 0.00317 | 0.001824 |
| 0.003168 | 0.001835 |
| 0.003166 | 0.001847 |
| 0.003164 | 0.001858 |
| 0.003162 | 0.00187 |
| 0.00316 | 0.001881 |
| 0.003158 | 0.001893 |
| 0.003156 | 0.001904 |
| 0.003153 | 0.001916 |
| 0.003151 | 0.001928 |
| 0.003149 | 0.001939 |
| 0.003147 | 0.001951 |
| 0.003145 | 0.001963 |
| 0.003143 | 0.001975 |
| 0.00314 | 0.001986 |
| 0.003138 | 0.001998 |
| 0.003136 | 0.00201 |
| 0.003134 | 0.002022 |
| 0.003131 | 0.002034 |
| 0.003129 | 0.002046 |
| 0.003127 | 0.002057 |
| 0.003124 | 0.002069 |
| 0.003122 | 0.002081 |
| 0.00312 | 0.002093 |
| 0.003117 | 0.002105 |
| 0.003115 | 0.002117 |
| 0.003112 | 0.002129 |
| 0.00311 | 0.002141 |
| 0.003107 | 0.002153 |
| 0.003105 | 0.002165 |
| 0.003102 | 0.002178 |
| 0.0031 | 0.00219 |
| 0.003097 | 0.002202 |
| 0.003095 | 0.002214 |
| 0.003092 | 0.002226 |
| 0.00309 | 0.002239 |
| 0.003087 | 0.002251 |
| 0.003084 | 0.002263 |
| 0.003082 | 0.002275 |
| 0.003079 | 0.002288 |
| 0.003076 | 0.0023 |
| 0.003074 | 0.002312 |
| 0.003071 | 0.002325 |

| | |
|---|---|
| 0.003068 | 0.002337 |
| 0.003065 | 0.00235 |
| 0.003063 | 0.002362 |
| 0.00306 | 0.002375 |
| 0.003057 | 0.002387 |
| 0.003054 | 0.0024 |
| 0.003051 | 0.002413 |
| 0.003049 | 0.002425 |
| 0.003046 | 0.002438 |
| 0.003043 | 0.00245 |
| 0.00304 | 0.002463 |
| 0.003037 | 0.002476 |
| 0.003034 | 0.002489 |
| 0.003031 | 0.002502 |
| 0.003028 | 0.002514 |
| 0.003025 | 0.002527 |
| 0.003022 | 0.00254 |
| 0.003019 | 0.002553 |
| 0.003016 | 0.002566 |
| 0.003013 | 0.002579 |
| 0.003009 | 0.002592 |
| 0.003006 | 0.002605 |
| 0.003003 | 0.002618 |
| 0.003 | 0.002631 |
| 0.002997 | 0.002644 |
| 0.002993 | 0.002657 |
| 0.00299 | 0.002671 |
| 0.002987 | 0.002684 |
| 0.002984 | 0.002697 |
| 0.00298 | 0.00271 |
| 0.002977 | 0.002724 |
| 0.002974 | 0.002737 |
| 0.00297 | 0.00275 |
| 0.002967 | 0.002764 |
| 0.002963 | 0.002777 |
| 0.00296 | 0.002791 |
| 0.002956 | 0.002804 |
| 0.002953 | 0.002818 |
| 0.002949 | 0.002831 |
| 0.002946 | 0.002845 |
| 0.002942 | 0.002859 |
| 0.002939 | 0.002872 |
| 0.002935 | 0.002886 |
| 0.002931 | 0.0029 |
| 0.002928 | 0.002914 |
| 0.002924 | 0.002927 |
| 0.00292 | 0.002941 |
| 0.002916 | 0.002955 |
| 0.002913 | 0.002969 |

| | |
|---|---|
| 0.002909 | 0.002983 |
| 0.002905 | 0.002997 |
| 0.002901 | 0.003011 |
| 0.002897 | 0.003025 |
| 0.002893 | 0.003039 |
| 0.002889 | 0.003054 |
| 0.002885 | 0.003068 |
| 0.002882 | 0.003082 |
| 0.002877 | 0.003096 |
| 0.002873 | 0.003111 |
| 0.002869 | 0.003125 |
| 0.002865 | 0.003139 |
| 0.002861 | 0.003154 |
| 0.002857 | 0.003168 |
| 0.002853 | 0.003183 |
| 0.002849 | 0.003197 |
| 0.002844 | 0.003212 |
| 0.00284 | 0.003227 |
| 0.002836 | 0.003241 |
| 0.002832 | 0.003256 |
| 0.002827 | 0.003271 |
| 0.002823 | 0.003286 |
| 0.002819 | 0.0033 |
| 0.002814 | 0.003315 |
| 0.00281 | 0.00333 |
| 0.002805 | 0.003345 |
| 0.002801 | 0.00336 |
| 0.002796 | 0.003375 |
| 0.002792 | 0.003391 |
| 0.002787 | 0.003406 |
| 0.002782 | 0.003421 |
| 0.002778 | 0.003436 |
| 0.002773 | 0.003452 |
| 0.002768 | 0.003467 |
| 0.002763 | 0.003482 |
| 0.002759 | 0.003498 |
| 0.002754 | 0.003513 |
| 0.002749 | 0.003529 |
| 0.002744 | 0.003545 |
| 0.002739 | 0.00356 |
| 0.002734 | 0.003576 |
| 0.002729 | 0.003592 |
| 0.002724 | 0.003607 |
| 0.002719 | 0.003623 |
| 0.002714 | 0.003639 |
| 0.002709 | 0.003655 |
| 0.002704 | 0.003671 |
| 0.002699 | 0.003687 |
| 0.002693 | 0.003703 |

| | |
|---|---|
| 0.002688 | 0.00372 |
| 0.002683 | 0.003736 |
| 0.002677 | 0.003752 |
| 0.002672 | 0.003768 |
| 0.002667 | 0.003785 |
| 0.002661 | 0.003801 |
| 0.002656 | 0.003818 |
| 0.00265 | 0.003834 |
| 0.002645 | 0.003851 |
| 0.002639 | 0.003868 |
| 0.002634 | 0.003884 |
| 0.002628 | 0.003901 |
| 0.002622 | 0.003918 |
| 0.002616 | 0.003935 |
| 0.002611 | 0.003952 |
| 0.002605 | 0.003969 |
| 0.002599 | 0.003986 |
| 0.002593 | 0.004003 |
| 0.002587 | 0.00402 |
| 0.002581 | 0.004038 |
| 0.002575 | 0.004055 |
| 0.002569 | 0.004072 |
| 0.002563 | 0.00409 |
| 0.002557 | 0.004107 |
| 0.002551 | 0.004125 |
| 0.002544 | 0.004142 |
| 0.002538 | 0.00416 |
| 0.002532 | 0.004178 |
| 0.002525 | 0.004196 |
| 0.002519 | 0.004214 |
| 0.002513 | 0.004232 |
| 0.002506 | 0.00425 |
| 0.002499 | 0.004268 |
| 0.002493 | 0.004286 |
| 0.002486 | 0.004304 |
| 0.00248 | 0.004323 |
| 0.002473 | 0.004341 |
| 0.002466 | 0.00436 |
| 0.002459 | 0.004378 |
| 0.002452 | 0.004397 |
| 0.002445 | 0.004415 |
| 0.002439 | 0.004434 |
| 0.002431 | 0.004453 |
| 0.002424 | 0.004472 |
| 0.002417 | 0.004491 |
| 0.00241 | 0.00451 |
| 0.002403 | 0.004529 |
| 0.002396 | 0.004548 |
| 0.002388 | 0.004568 |

| | |
|---|---|
| 0.002381 | 0.004587 |
| 0.002374 | 0.004606 |
| 0.002366 | 0.004626 |
| 0.002359 | 0.004646 |
| 0.002351 | 0.004665 |
| 0.002343 | 0.004685 |
| 0.002336 | 0.004705 |
| 0.002328 | 0.004725 |
| 0.00232 | 0.004745 |
| 0.002312 | 0.004765 |
| 0.002304 | 0.004785 |
| 0.002296 | 0.004805 |
| 0.002288 | 0.004826 |
| 0.00228 | 0.004846 |
| 0.002272 | 0.004867 |
| 0.002264 | 0.004887 |
| 0.002256 | 0.004908 |
| 0.002247 | 0.004929 |
| 0.002239 | 0.00495 |
| 0.002231 | 0.004971 |
| 0.002222 | 0.004992 |
| 0.002213 | 0.005013 |
| 0.002205 | 0.005034 |
| 0.002196 | 0.005056 |
| 0.002187 | 0.005077 |
| 0.002179 | 0.005099 |
| 0.00217 | 0.00512 |
| 0.002161 | 0.005142 |
| 0.002152 | 0.005164 |
| 0.002143 | 0.005186 |
| 0.002133 | 0.005208 |
| 0.002124 | 0.00523 |
| 0.002115 | 0.005252 |
| 0.002106 | 0.005275 |
| 0.002096 | 0.005297 |
| 0.002087 | 0.00532 |
| 0.002077 | 0.005342 |
| 0.002067 | 0.005365 |
| 0.002058 | 0.005388 |
| 0.002048 | 0.005411 |
| 0.002038 | 0.005434 |
| 0.002028 | 0.005457 |
| 0.002018 | 0.005481 |
| 0.002008 | 0.005504 |
| 0.001998 | 0.005528 |
| 0.001988 | 0.005551 |
| 0.001977 | 0.005575 |
| 0.001967 | 0.005599 |
| 0.001956 | 0.005623 |

| | |
|---|---|
| 0.001946 | 0.005647 |
| 0.001935 | 0.005672 |
| 0.001924 | 0.005696 |
| 0.001914 | 0.005721 |
| 0.001903 | 0.005745 |
| 0.001892 | 0.00577 |
| 0.001881 | 0.005795 |
| 0.001869 | 0.00582 |
| 0.001858 | 0.005845 |
| 0.001847 | 0.00587 |
| 0.001835 | 0.005896 |
| 0.001824 | 0.005921 |
| 0.001812 | 0.005947 |
| 0.001801 | 0.005973 |
| 0.001789 | 0.005999 |
| 0.001777 | 0.006025 |
| 0.001765 | 0.006051 |
| 0.001753 | 0.006077 |
| 0.001741 | 0.006104 |
| 0.001728 | 0.006131 |
| 0.001716 | 0.006157 |
| 0.001704 | 0.006184 |
| 0.001691 | 0.006211 |
| 0.001678 | 0.006239 |
| 0.001666 | 0.006266 |
| 0.001653 | 0.006294 |
| 0.00164 | 0.006321 |
| 0.001627 | 0.006349 |
| 0.001613 | 0.006377 |
| 0.0016 | 0.006405 |
| 0.001587 | 0.006434 |
| 0.001573 | 0.006462 |
| 0.001559 | 0.006491 |
| 0.001546 | 0.006519 |
| 0.001532 | 0.006548 |
| 0.001518 | 0.006578 |
| 0.001504 | 0.006607 |
| 0.00149 | 0.006636 |
| 0.001475 | 0.006666 |
| 0.001461 | 0.006696 |
| 0.001446 | 0.006726 |
| 0.001431 | 0.006756 |
| 0.001417 | 0.006786 |
| 0.001402 | 0.006817 |
| 0.001387 | 0.006848 |
| 0.001371 | 0.006878 |
| 0.001356 | 0.00691 |
| 0.001341 | 0.006941 |
| 0.001325 | 0.006972 |

| | |
|---|---|
| 0.001309 | 0.007004 |
| 0.001293 | 0.007036 |
| 0.001277 | 0.007068 |
| 0.001261 | 0.0071 |
| 0.001245 | 0.007133 |
| 0.001228 | 0.007165 |
| 0.001212 | 0.007198 |
| 0.001195 | 0.007231 |
| 0.001178 | 0.007264 |
| 0.001161 | 0.007298 |
| 0.001144 | 0.007332 |
| 0.001127 | 0.007365 |
| 0.001109 | 0.0074 |
| 0.001092 | 0.007434 |
| 0.001074 | 0.007468 |
| 0.001056 | 0.007503 |
| 0.001038 | 0.007538 |
| 0.001019 | 0.007574 |
| 0.001001 | 0.007609 |
| 0.000982 | 0.007645 |
| 0.000964 | 0.007681 |
| 0.000945 | 0.007717 |
| 0.000925 | 0.007753 |
| 0.000906 | 0.00779 |
| 0.000887 | 0.007827 |
| 0.000867 | 0.007864 |
| 0.000847 | 0.007902 |
| 0.000827 | 0.007939 |
| 0.000807 | 0.007977 |
| 0.000786 | 0.008016 |
| 0.000766 | 0.008054 |
| 0.000745 | 0.008093 |
| 0.000724 | 0.008132 |
| 0.000703 | 0.008171 |
| 0.000681 | 0.008211 |
| 0.00066 | 0.008251 |
| 0.000638 | 0.008291 |
| 0.000616 | 0.008331 |
| 0.000594 | 0.008372 |
| 0.000571 | 0.008413 |
| 0.000549 | 0.008454 |
| 0.000526 | 0.008496 |
| 0.000503 | 0.008538 |
| 0.000479 | 0.00858 |
| 0.000456 | 0.008623 |
| 0.000432 | 0.008666 |
| 0.000408 | 0.008709 |
| 0.000384 | 0.008753 |
| 0.000359 | 0.008796 |

| | |
|---|---|
| 0.000334 | 0.008841 |
| 0.000309 | 0.008885 |
| 0.000284 | 0.00893 |
| 0.000259 | 0.008975 |
| 0.000233 | 0.009021 |
| 0.000207 | 0.009067 |
| 0.00018 | 0.009113 |
| 0.000154 | 0.00916 |
| 0.000127 | 0.009207 |
| 9.99E-05 | 0.009254 |
| 7.25E-05 | 0.009302 |
| 4.48E-05 | 0.00935 |
| 1.69E-05 | 0.009399 |
| -1.1E-05 | 0.009448 |
| -4E-05 | 0.009497 |
| -6.9E-05 | 0.009547 |
| -9.8E-05 | 0.009597 |
| -0.00013 | 0.009648 |
| -0.00016 | 0.009699 |
| -0.00019 | 0.00975 |
| -0.00022 | 0.009802 |
| -0.00025 | 0.009854 |
| -0.00028 | 0.009907 |
| -0.00031 | 0.00996 |
| -0.00034 | 0.010014 |
| -0.00037 | 0.010068 |
| -0.00041 | 0.010122 |
| -0.00044 | 0.010177 |
| -0.00047 | 0.010233 |
| -0.00051 | 0.010289 |
| -0.00054 | 0.010345 |
| -0.00057 | 0.010402 |
| -0.00061 | 0.01046 |
| -0.00064 | 0.010518 |
| -0.00068 | 0.010576 |
| -0.00072 | 0.010635 |
| -0.00075 | 0.010695 |
| -0.00079 | 0.010755 |
| -0.00083 | 0.010816 |
| -0.00086 | 0.010877 |
| -0.0009 | 0.010939 |
| -0.00094 | 0.011001 |
| -0.00098 | 0.011064 |
| -0.00102 | 0.011128 |
| -0.00106 | 0.011192 |
| -0.0011 | 0.011257 |
| -0.00114 | 0.011322 |
| -0.00118 | 0.011388 |
| -0.00122 | 0.011455 |

| | |
|---|---|
| -0.00126 | 0.011522 |
| -0.00131 | 0.01159 |
| -0.00135 | 0.011659 |
| -0.00139 | 0.011728 |
| -0.00144 | 0.011798 |
| -0.00148 | 0.011869 |
| -0.00153 | 0.01194 |
| -0.00157 | 0.012012 |
| -0.00162 | 0.012085 |
| -0.00167 | 0.012158 |
| -0.00171 | 0.012233 |
| -0.00176 | 0.012308 |
| -0.00181 | 0.012384 |
| -0.00186 | 0.01246 |
| -0.00191 | 0.012538 |
| -0.00196 | 0.012616 |
| -0.00201 | 0.012695 |
| -0.00206 | 0.012775 |
| -0.00212 | 0.012856 |
| -0.00217 | 0.012938 |
| -0.00222 | 0.01302 |
| -0.00228 | 0.013104 |
| -0.00233 | 0.013188 |
| -0.00239 | 0.013273 |
| -0.00245 | 0.01336 |
| -0.00251 | 0.013447 |
| -0.00256 | 0.013535 |
| -0.00262 | 0.013624 |
| -0.00268 | 0.013714 |
| -0.00274 | 0.013806 |
| -0.00281 | 0.013898 |
| -0.00287 | 0.013991 |
| -0.00293 | 0.014086 |
| -0.003 | 0.014181 |
| -0.00306 | 0.014278 |
| -0.00313 | 0.014375 |
| -0.00319 | 0.014474 |
| -0.00326 | 0.014574 |
| -0.00333 | 0.014676 |
| -0.0034 | 0.014778 |
| -0.00347 | 0.014882 |
| -0.00354 | 0.014987 |
| -0.00362 | 0.015094 |
| -0.00369 | 0.015201 |
| -0.00377 | 0.01531 |
| -0.00384 | 0.015421 |
| -0.00392 | 0.015532 |
| -0.004 | 0.015646 |
| -0.00408 | 0.01576 |

| | |
|---|---|
| -0.00416 | 0.015876 |
| -0.00424 | 0.015994 |
| -0.00432 | 0.016113 |
| -0.00441 | 0.016234 |
| -0.0045 | 0.016356 |
| -0.00458 | 0.01648 |
| -0.00467 | 0.016606 |
| -0.00476 | 0.016733 |
| -0.00485 | 0.016862 |
| -0.00495 | 0.016993 |
| -0.00504 | 0.017126 |
| -0.00514 | 0.01726 |
| -0.00523 | 0.017396 |
| -0.00533 | 0.017535 |
| -0.00543 | 0.017675 |
| -0.00553 | 0.017817 |
| -0.00564 | 0.017961 |
| -0.00574 | 0.018107 |
| -0.00585 | 0.018256 |
| -0.00596 | 0.018407 |
| -0.00607 | 0.018559 |
| -0.00619 | 0.018714 |
| -0.0063 | 0.018872 |
| -0.00642 | 0.019032 |
| -0.00654 | 0.019194 |
| -0.00666 | 0.019358 |
| -0.00678 | 0.019526 |
| -0.00691 | 0.019695 |
| -0.00703 | 0.019868 |
| -0.00716 | 0.020043 |
| -0.00729 | 0.020221 |
| -0.00743 | 0.020402 |
| -0.00757 | 0.020585 |
| -0.00771 | 0.020772 |
| -0.00785 | 0.020962 |
| -0.00799 | 0.021155 |
| -0.00814 | 0.021351 |
| -0.00829 | 0.02155 |
| -0.00844 | 0.021753 |
| -0.0086 | 0.021959 |
| -0.00876 | 0.022168 |
| -0.00892 | 0.022382 |
| -0.00908 | 0.022599 |
| -0.00925 | 0.022819 |
| -0.00942 | 0.023044 |
| -0.0096 | 0.023273 |
| -0.00978 | 0.023506 |
| -0.00996 | 0.023743 |
| -0.01015 | 0.023984 |

| | |
|---|---|
| -0.01034 | 0.02423 |
| -0.01053 | 0.02448 |
| -0.01073 | 0.024735 |
| -0.01093 | 0.024995 |
| -0.01114 | 0.02526 |
| -0.01135 | 0.02553 |
| -0.01156 | 0.025805 |
| -0.01178 | 0.026086 |
| -0.01201 | 0.026372 |
| -0.01224 | 0.026664 |
| -0.01247 | 0.026962 |
| -0.01271 | 0.027265 |
| -0.01296 | 0.027575 |
| -0.01321 | 0.027892 |
| -0.01346 | 0.028215 |
| -0.01373 | 0.028545 |
| -0.01399 | 0.028882 |
| -0.01427 | 0.029226 |
| -0.01455 | 0.029577 |
| -0.01484 | 0.029937 |
| -0.01514 | 0.030304 |
| -0.01544 | 0.030679 |
| -0.01575 | 0.031063 |
| -0.01607 | 0.031456 |
| -0.01639 | 0.031858 |
| -0.01673 | 0.032269 |
| -0.01707 | 0.03269 |
| -0.01742 | 0.03312 |
| -0.01778 | 0.033562 |
| -0.01815 | 0.034013 |
| -0.01853 | 0.034476 |
| -0.01892 | 0.034951 |
| -0.01932 | 0.035437 |
| -0.01973 | 0.035936 |
| -0.02016 | 0.036447 |
| -0.02059 | 0.036972 |
| -0.02104 | 0.037511 |
| -0.0215 | 0.038063 |
| -0.02198 | 0.038631 |
| -0.02246 | 0.039214 |
| -0.02297 | 0.039814 |
| -0.02348 | 0.040429 |
| -0.02402 | 0.041063 |
| -0.02456 | 0.041714 |
| -0.02513 | 0.042384 |
| -0.02571 | 0.043074 |
| -0.02632 | 0.043784 |
| -0.02694 | 0.044516 |
| -0.02758 | 0.04527 |

| | |
|---|---|
| -0.02824 | 0.046047 |
| -0.02893 | 0.046848 |
| -0.02964 | 0.047675 |
| -0.03037 | 0.048528 |
| -0.03113 | 0.049409 |
| -0.03191 | 0.05032 |
| -0.03273 | 0.05126 |
| -0.03357 | 0.052233 |
| -0.03444 | 0.053239 |
| -0.03535 | 0.054281 |
| -0.03629 | 0.055359 |
| -0.03726 | 0.056477 |
| -0.03827 | 0.057635 |
| -0.03933 | 0.058836 |
| -0.04042 | 0.060083 |
| -0.04156 | 0.061378 |
| -0.04275 | 0.062724 |
| -0.04398 | 0.064123 |
| -0.04527 | 0.065578 |
| -0.04661 | 0.067094 |
| -0.04802 | 0.068672 |
| -0.04948 | 0.070319 |
| -0.05102 | 0.072036 |
| -0.05262 | 0.07383 |
| -0.0543 | 0.075704 |
| -0.05606 | 0.077664 |
| -0.0579 | 0.079716 |
| -0.05984 | 0.081866 |
| -0.06188 | 0.084121 |
| -0.06402 | 0.086488 |
| -0.06627 | 0.088975 |
| -0.06865 | 0.091591 |
| -0.07116 | 0.094346 |
| -0.07381 | 0.097251 |
| -0.07661 | 0.100316 |
| -0.07958 | 0.103556 |
| -0.08272 | 0.106985 |
| -0.08606 | 0.110617 |
| -0.08962 | 0.114472 |
| -0.0934 | 0.118569 |
| -0.09743 | 0.122929 |
| -0.10174 | 0.127578 |
| -0.10635 | 0.132544 |
| -0.1113 | 0.137857 |
| -0.11661 | 0.143552 |
| -0.12233 | 0.149671 |
| -0.1285 | 0.156259 |
| -0.13516 | 0.163368 |
| -0.14239 | 0.171058 |

| | |
|---|---|
| -0.15025 | 0.179399 |
| -0.15881 | 0.188472 |
| -0.16817 | 0.198369 |
| -0.17844 | 0.209201 |
| -0.18973 | 0.221098 |
| -0.20221 | 0.234212 |
| -0.21605 | 0.248727 |
| -0.23146 | 0.264863 |
| -0.24872 | 0.282888 |
| -0.26813 | 0.303128 |
| -0.29009 | 0.325985 |
| -0.31511 | 0.351964 |
| -0.3438 | 0.381697 |
| -0.37696 | 0.415996 |
| -0.41562 | 0.455912 |
| -0.46116 | 0.502828 |
| -0.51541 | 0.558601 |
| -0.58087 | 0.625772 |
| -0.66108 | 0.707908 |
| -0.76112 | 0.810148 |
| -0.88858 | 0.940153 |
| -1.05524 | 1.1098 |
| -1.28027 | 1.3384 |
| -1.59682 | 1.65933 |
| -2.06687 | 2.13493 |
| -2.81912 | 2.89451 |
| -4.16375 | 4.24952 |
| -7.04459 | 7.1467 |
| -15.8728 | 16.0065 |
| -104.763 | 105.013 |

**Table S1-A2.** Keypoints used for generating an hyperbolic notch with opening angle $2\alpha$ = 60 degrees and radius of the notch tip equal to 0.01mm.

| X | Y |
|---|---|
| 0.004 | 0 |
| 0.004 | 0.000012 |
| 0.004 | 2.4E-05 |
| 0.004 | 3.6E-05 |
| 0.004 | 4.8E-05 |
| 0.004 | 6E-05 |
| 0.004 | 7.2E-05 |
| 0.004 | 8.4E-05 |
| 0.004 | 9.6E-05 |
| 0.003999 | 0.000108 |
| 0.003999 | 0.00012 |
| 0.003999 | 0.000132 |
| 0.003999 | 0.000144 |
| 0.003999 | 0.000156 |

| | |
|---|---|
| 0.003999 | 0.000168 |
| 0.003998 | 0.00018 |
| 0.003998 | 0.000192 |
| 0.003998 | 0.000204 |
| 0.003998 | 0.000216 |
| 0.003997 | 0.000228 |
| 0.003997 | 0.00024 |
| 0.003997 | 0.000252 |
| 0.003997 | 0.000264 |
| 0.003996 | 0.000276 |
| 0.003996 | 0.000288 |
| 0.003995 | 0.0003 |
| 0.003995 | 0.000312 |
| 0.003995 | 0.000324 |
| 0.003994 | 0.000336 |
| 0.003994 | 0.000348 |
| 0.003994 | 0.00036 |
| 0.003993 | 0.000372 |
| 0.003993 | 0.000384 |
| 0.003992 | 0.000397 |
| 0.003992 | 0.000409 |
| 0.003991 | 0.000421 |
| 0.003991 | 0.000433 |
| 0.00399 | 0.000445 |
| 0.00399 | 0.000457 |
| 0.003989 | 0.000469 |
| 0.003988 | 0.000481 |
| 0.003988 | 0.000493 |
| 0.003987 | 0.000505 |
| 0.003987 | 0.000517 |
| 0.003986 | 0.000529 |
| 0.003985 | 0.000541 |
| 0.003985 | 0.000553 |
| 0.003984 | 0.000566 |
| 0.003983 | 0.000578 |
| 0.003983 | 0.00059 |
| 0.003982 | 0.000602 |
| 0.003981 | 0.000614 |
| 0.00398 | 0.000626 |
| 0.00398 | 0.000638 |
| 0.003979 | 0.00065 |
| 0.003978 | 0.000662 |
| 0.003977 | 0.000675 |
| 0.003976 | 0.000687 |
| 0.003976 | 0.000699 |
| 0.003975 | 0.000711 |
| 0.003974 | 0.000723 |
| 0.003973 | 0.000735 |
| 0.003972 | 0.000747 |

| | |
|---|---|
| 0.003971 | 0.00076 |
| 0.00397 | 0.000772 |
| 0.003969 | 0.000784 |
| 0.003968 | 0.000796 |
| 0.003967 | 0.000808 |
| 0.003966 | 0.000821 |
| 0.003965 | 0.000833 |
| 0.003964 | 0.000845 |
| 0.003963 | 0.000857 |
| 0.003962 | 0.000869 |
| 0.003961 | 0.000882 |
| 0.00396 | 0.000894 |
| 0.003959 | 0.000906 |
| 0.003958 | 0.000918 |
| 0.003957 | 0.000931 |
| 0.003956 | 0.000943 |
| 0.003954 | 0.000955 |
| 0.003953 | 0.000967 |
| 0.003952 | 0.00098 |
| 0.003951 | 0.000992 |
| 0.00395 | 0.001004 |
| 0.003948 | 0.001017 |
| 0.003947 | 0.001029 |
| 0.003946 | 0.001041 |
| 0.003945 | 0.001054 |
| 0.003943 | 0.001066 |
| 0.003942 | 0.001078 |
| 0.003941 | 0.001091 |
| 0.003939 | 0.001103 |
| 0.003938 | 0.001115 |
| 0.003937 | 0.001128 |
| 0.003935 | 0.00114 |
| 0.003934 | 0.001153 |
| 0.003932 | 0.001165 |
| 0.003931 | 0.001177 |
| 0.003929 | 0.00119 |
| 0.003928 | 0.001202 |
| 0.003927 | 0.001215 |
| 0.003925 | 0.001227 |
| 0.003923 | 0.00124 |
| 0.003922 | 0.001252 |
| 0.00392 | 0.001264 |
| 0.003919 | 0.001277 |
| 0.003917 | 0.001289 |
| 0.003916 | 0.001302 |
| 0.003914 | 0.001314 |
| 0.003912 | 0.001327 |
| 0.003911 | 0.00134 |
| 0.003909 | 0.001352 |

| | |
|---|---|
| 0.003907 | 0.001365 |
| 0.003906 | 0.001377 |
| 0.003904 | 0.00139 |
| 0.003902 | 0.001402 |
| 0.0039 | 0.001415 |
| 0.003899 | 0.001428 |
| 0.003897 | 0.00144 |
| 0.003895 | 0.001453 |
| 0.003893 | 0.001465 |
| 0.003891 | 0.001478 |
| 0.00389 | 0.001491 |
| 0.003888 | 0.001503 |
| 0.003886 | 0.001516 |
| 0.003884 | 0.001529 |
| 0.003882 | 0.001541 |
| 0.00388 | 0.001554 |
| 0.003878 | 0.001567 |
| 0.003876 | 0.00158 |
| 0.003874 | 0.001592 |
| 0.003872 | 0.001605 |
| 0.00387 | 0.001618 |
| 0.003868 | 0.001631 |
| 0.003866 | 0.001644 |
| 0.003864 | 0.001656 |
| 0.003862 | 0.001669 |
| 0.00386 | 0.001682 |
| 0.003857 | 0.001695 |
| 0.003855 | 0.001708 |
| 0.003853 | 0.001721 |
| 0.003851 | 0.001734 |
| 0.003849 | 0.001746 |
| 0.003846 | 0.001759 |
| 0.003844 | 0.001772 |
| 0.003842 | 0.001785 |
| 0.00384 | 0.001798 |
| 0.003837 | 0.001811 |
| 0.003835 | 0.001824 |
| 0.003833 | 0.001837 |
| 0.00383 | 0.00185 |
| 0.003828 | 0.001863 |
| 0.003825 | 0.001876 |
| 0.003823 | 0.001889 |
| 0.003821 | 0.001902 |
| 0.003818 | 0.001916 |
| 0.003816 | 0.001929 |
| 0.003813 | 0.001942 |
| 0.003811 | 0.001955 |
| 0.003808 | 0.001968 |
| 0.003806 | 0.001981 |

| | |
|---|---|
| 0.003803 | 0.001994 |
| 0.0038 | 0.002008 |
| 0.003798 | 0.002021 |
| 0.003795 | 0.002034 |
| 0.003793 | 0.002047 |
| 0.00379 | 0.002061 |
| 0.003787 | 0.002074 |
| 0.003784 | 0.002087 |
| 0.003782 | 0.0021 |
| 0.003779 | 0.002114 |
| 0.003776 | 0.002127 |
| 0.003773 | 0.002141 |
| 0.003771 | 0.002154 |
| 0.003768 | 0.002167 |
| 0.003765 | 0.002181 |
| 0.003762 | 0.002194 |
| 0.003759 | 0.002208 |
| 0.003756 | 0.002221 |
| 0.003753 | 0.002235 |
| 0.00375 | 0.002248 |
| 0.003747 | 0.002262 |
| 0.003744 | 0.002275 |
| 0.003741 | 0.002289 |
| 0.003738 | 0.002302 |
| 0.003735 | 0.002316 |
| 0.003732 | 0.00233 |
| 0.003729 | 0.002343 |
| 0.003726 | 0.002357 |
| 0.003723 | 0.00237 |
| 0.00372 | 0.002384 |
| 0.003716 | 0.002398 |
| 0.003713 | 0.002412 |
| 0.00371 | 0.002425 |
| 0.003707 | 0.002439 |
| 0.003703 | 0.002453 |
| 0.0037 | 0.002467 |
| 0.003697 | 0.002481 |
| 0.003694 | 0.002494 |
| 0.00369 | 0.002508 |
| 0.003687 | 0.002522 |
| 0.003683 | 0.002536 |
| 0.00368 | 0.00255 |
| 0.003676 | 0.002564 |
| 0.003673 | 0.002578 |
| 0.003669 | 0.002592 |
| 0.003666 | 0.002606 |
| 0.003662 | 0.00262 |
| 0.003659 | 0.002634 |
| 0.003655 | 0.002648 |

| | |
|---|---|
| 0.003652 | 0.002662 |
| 0.003648 | 0.002677 |
| 0.003644 | 0.002691 |
| 0.003641 | 0.002705 |
| 0.003637 | 0.002719 |
| 0.003633 | 0.002733 |
| 0.003629 | 0.002748 |
| 0.003626 | 0.002762 |
| 0.003622 | 0.002776 |
| 0.003618 | 0.00279 |
| 0.003614 | 0.002805 |
| 0.00361 | 0.002819 |
| 0.003606 | 0.002834 |
| 0.003602 | 0.002848 |
| 0.003598 | 0.002862 |
| 0.003594 | 0.002877 |
| 0.00359 | 0.002891 |
| 0.003586 | 0.002906 |
| 0.003582 | 0.00292 |
| 0.003578 | 0.002935 |
| 0.003574 | 0.00295 |
| 0.00357 | 0.002964 |
| 0.003566 | 0.002979 |
| 0.003561 | 0.002994 |
| 0.003557 | 0.003008 |
| 0.003553 | 0.003023 |
| 0.003549 | 0.003038 |
| 0.003544 | 0.003053 |
| 0.00354 | 0.003067 |
| 0.003536 | 0.003082 |
| 0.003531 | 0.003097 |
| 0.003527 | 0.003112 |
| 0.003522 | 0.003127 |
| 0.003518 | 0.003142 |
| 0.003513 | 0.003157 |
| 0.003509 | 0.003172 |
| 0.003504 | 0.003187 |
| 0.0035 | 0.003202 |
| 0.003495 | 0.003217 |
| 0.00349 | 0.003232 |
| 0.003486 | 0.003247 |
| 0.003481 | 0.003262 |
| 0.003476 | 0.003278 |
| 0.003471 | 0.003293 |
| 0.003467 | 0.003308 |
| 0.003462 | 0.003323 |
| 0.003457 | 0.003339 |
| 0.003452 | 0.003354 |
| 0.003447 | 0.00337 |

| | |
|---|---|
| 0.003442 | 0.003385 |
| 0.003437 | 0.0034 |
| 0.003432 | 0.003416 |
| 0.003427 | 0.003431 |
| 0.003422 | 0.003447 |
| 0.003417 | 0.003463 |
| 0.003412 | 0.003478 |
| 0.003407 | 0.003494 |
| 0.003402 | 0.003509 |
| 0.003396 | 0.003525 |
| 0.003391 | 0.003541 |
| 0.003386 | 0.003557 |
| 0.003381 | 0.003573 |
| 0.003375 | 0.003588 |
| 0.00337 | 0.003604 |
| 0.003364 | 0.00362 |
| 0.003359 | 0.003636 |
| 0.003353 | 0.003652 |
| 0.003348 | 0.003668 |
| 0.003342 | 0.003684 |
| 0.003337 | 0.0037 |
| 0.003331 | 0.003716 |
| 0.003326 | 0.003733 |
| 0.00332 | 0.003749 |
| 0.003314 | 0.003765 |
| 0.003308 | 0.003781 |
| 0.003303 | 0.003798 |
| 0.003297 | 0.003814 |
| 0.003291 | 0.00383 |
| 0.003285 | 0.003847 |
| 0.003279 | 0.003863 |
| 0.003273 | 0.00388 |
| 0.003267 | 0.003896 |
| 0.003261 | 0.003913 |
| 0.003255 | 0.003929 |
| 0.003249 | 0.003946 |
| 0.003243 | 0.003963 |
| 0.003236 | 0.003979 |
| 0.00323 | 0.003996 |
| 0.003224 | 0.004013 |
| 0.003218 | 0.00403 |
| 0.003211 | 0.004047 |
| 0.003205 | 0.004064 |
| 0.003198 | 0.004081 |
| 0.003192 | 0.004098 |
| 0.003186 | 0.004115 |
| 0.003179 | 0.004132 |
| 0.003172 | 0.004149 |
| 0.003166 | 0.004166 |

| | |
|---|---|
| 0.003159 | 0.004183 |
| 0.003152 | 0.004201 |
| 0.003146 | 0.004218 |
| 0.003139 | 0.004235 |
| 0.003132 | 0.004253 |
| 0.003125 | 0.00427 |
| 0.003118 | 0.004288 |
| 0.003111 | 0.004305 |
| 0.003104 | 0.004323 |
| 0.003097 | 0.00434 |
| 0.00309 | 0.004358 |
| 0.003083 | 0.004376 |
| 0.003076 | 0.004394 |
| 0.003069 | 0.004411 |
| 0.003061 | 0.004429 |
| 0.003054 | 0.004447 |
| 0.003047 | 0.004465 |
| 0.003039 | 0.004483 |
| 0.003032 | 0.004501 |
| 0.003025 | 0.004519 |
| 0.003017 | 0.004537 |
| 0.003009 | 0.004556 |
| 0.003002 | 0.004574 |
| 0.002994 | 0.004592 |
| 0.002986 | 0.00461 |
| 0.002979 | 0.004629 |
| 0.002971 | 0.004647 |
| 0.002963 | 0.004666 |
| 0.002955 | 0.004684 |
| 0.002947 | 0.004703 |
| 0.002939 | 0.004722 |
| 0.002931 | 0.00474 |
| 0.002923 | 0.004759 |
| 0.002915 | 0.004778 |
| 0.002907 | 0.004797 |
| 0.002898 | 0.004816 |
| 0.00289 | 0.004835 |
| 0.002882 | 0.004854 |
| 0.002873 | 0.004873 |
| 0.002865 | 0.004892 |
| 0.002856 | 0.004911 |
| 0.002848 | 0.00493 |
| 0.002839 | 0.00495 |
| 0.002831 | 0.004969 |
| 0.002822 | 0.004989 |
| 0.002813 | 0.005008 |
| 0.002804 | 0.005028 |
| 0.002795 | 0.005047 |
| 0.002786 | 0.005067 |

| | |
|---|---|
| 0.002777 | 0.005087 |
| 0.002768 | 0.005106 |
| 0.002759 | 0.005126 |
| 0.00275 | 0.005146 |
| 0.002741 | 0.005166 |
| 0.002732 | 0.005186 |
| 0.002722 | 0.005206 |
| 0.002713 | 0.005226 |
| 0.002703 | 0.005247 |
| 0.002694 | 0.005267 |
| 0.002684 | 0.005287 |
| 0.002675 | 0.005308 |
| 0.002665 | 0.005328 |
| 0.002655 | 0.005349 |
| 0.002645 | 0.005369 |
| 0.002636 | 0.00539 |
| 0.002626 | 0.005411 |
| 0.002616 | 0.005431 |
| 0.002606 | 0.005452 |
| 0.002595 | 0.005473 |
| 0.002585 | 0.005494 |
| 0.002575 | 0.005515 |
| 0.002565 | 0.005537 |
| 0.002554 | 0.005558 |
| 0.002544 | 0.005579 |
| 0.002533 | 0.0056 |
| 0.002523 | 0.005622 |
| 0.002512 | 0.005643 |
| 0.002501 | 0.005665 |
| 0.00249 | 0.005687 |
| 0.00248 | 0.005708 |
| 0.002469 | 0.00573 |
| 0.002458 | 0.005752 |
| 0.002447 | 0.005774 |
| 0.002435 | 0.005796 |
| 0.002424 | 0.005818 |
| 0.002413 | 0.00584 |
| 0.002402 | 0.005862 |
| 0.00239 | 0.005885 |
| 0.002379 | 0.005907 |
| 0.002367 | 0.00593 |
| 0.002355 | 0.005952 |
| 0.002344 | 0.005975 |
| 0.002332 | 0.005998 |
| 0.00232 | 0.006021 |
| 0.002308 | 0.006043 |
| 0.002296 | 0.006066 |
| 0.002284 | 0.006089 |
| 0.002272 | 0.006113 |

| | |
|---|---|
| 0.002259 | 0.006136 |
| 0.002247 | 0.006159 |
| 0.002234 | 0.006183 |
| 0.002222 | 0.006206 |
| 0.002209 | 0.00623 |
| 0.002197 | 0.006253 |
| 0.002184 | 0.006277 |
| 0.002171 | 0.006301 |
| 0.002158 | 0.006325 |
| 0.002145 | 0.006349 |
| 0.002132 | 0.006373 |
| 0.002119 | 0.006397 |
| 0.002105 | 0.006422 |
| 0.002092 | 0.006446 |
| 0.002078 | 0.00647 |
| 0.002065 | 0.006495 |
| 0.002051 | 0.00652 |
| 0.002037 | 0.006544 |
| 0.002023 | 0.006569 |
| 0.00201 | 0.006594 |
| 0.001995 | 0.006619 |
| 0.001981 | 0.006645 |
| 0.001967 | 0.00667 |
| 0.001953 | 0.006695 |
| 0.001938 | 0.006721 |
| 0.001924 | 0.006746 |
| 0.001909 | 0.006772 |
| 0.001894 | 0.006798 |
| 0.00188 | 0.006824 |
| 0.001865 | 0.00685 |
| 0.00185 | 0.006876 |
| 0.001835 | 0.006902 |
| 0.001819 | 0.006928 |
| 0.001804 | 0.006955 |
| 0.001788 | 0.006981 |
| 0.001773 | 0.007008 |
| 0.001757 | 0.007035 |
| 0.001741 | 0.007061 |
| 0.001726 | 0.007088 |
| 0.00171 | 0.007115 |
| 0.001693 | 0.007143 |
| 0.001677 | 0.00717 |
| 0.001661 | 0.007197 |
| 0.001644 | 0.007225 |
| 0.001628 | 0.007253 |
| 0.001611 | 0.00728 |
| 0.001594 | 0.007308 |
| 0.001577 | 0.007336 |
| 0.00156 | 0.007365 |

| | |
|---|---|
| 0.001543 | 0.007393 |
| 0.001526 | 0.007421 |
| 0.001509 | 0.00745 |
| 0.001491 | 0.007479 |
| 0.001473 | 0.007507 |
| 0.001456 | 0.007536 |
| 0.001438 | 0.007565 |
| 0.00142 | 0.007594 |
| 0.001401 | 0.007624 |
| 0.001383 | 0.007653 |
| 0.001365 | 0.007683 |
| 0.001346 | 0.007713 |
| 0.001327 | 0.007742 |
| 0.001309 | 0.007772 |
| 0.00129 | 0.007803 |
| 0.00127 | 0.007833 |
| 0.001251 | 0.007863 |
| 0.001232 | 0.007894 |
| 0.001212 | 0.007924 |
| 0.001193 | 0.007955 |
| 0.001173 | 0.007986 |
| 0.001153 | 0.008017 |
| 0.001133 | 0.008049 |
| 0.001112 | 0.00808 |
| 0.001092 | 0.008112 |
| 0.001071 | 0.008144 |
| 0.001051 | 0.008175 |
| 0.00103 | 0.008207 |
| 0.001009 | 0.00824 |
| 0.000987 | 0.008272 |
| 0.000966 | 0.008305 |
| 0.000944 | 0.008337 |
| 0.000923 | 0.00837 |
| 0.000901 | 0.008403 |
| 0.000879 | 0.008436 |
| 0.000857 | 0.00847 |
| 0.000834 | 0.008503 |
| 0.000812 | 0.008537 |
| 0.000789 | 0.008571 |
| 0.000766 | 0.008605 |
| 0.000743 | 0.008639 |
| 0.00072 | 0.008673 |
| 0.000696 | 0.008708 |
| 0.000673 | 0.008743 |
| 0.000649 | 0.008778 |
| 0.000625 | 0.008813 |
| 0.000601 | 0.008848 |
| 0.000576 | 0.008883 |
| 0.000552 | 0.008919 |

| | |
|---|---|
| 0.000527 | 0.008955 |
| 0.000502 | 0.008991 |
| 0.000477 | 0.009027 |
| 0.000452 | 0.009064 |
| 0.000426 | 0.0091 |
| 0.0004 | 0.009137 |
| 0.000374 | 0.009174 |
| 0.000348 | 0.009211 |
| 0.000322 | 0.009249 |
| 0.000295 | 0.009286 |
| 0.000269 | 0.009324 |
| 0.000242 | 0.009362 |
| 0.000214 | 0.0094 |
| 0.000187 | 0.009439 |
| 0.000159 | 0.009478 |
| 0.000131 | 0.009517 |
| 0.000103 | 0.009556 |
| 7.48E-05 | 0.009595 |
| 4.62E-05 | 0.009635 |
| 1.74E-05 | 0.009674 |
| -1.2E-05 | 0.009714 |
| -4.1E-05 | 0.009755 |
| -7.1E-05 | 0.009795 |
| -0.0001 | 0.009836 |
| -0.00013 | 0.009877 |
| -0.00016 | 0.009918 |
| -0.00019 | 0.009959 |
| -0.00022 | 0.010001 |
| -0.00025 | 0.010043 |
| -0.00028 | 0.010085 |
| -0.00032 | 0.010128 |
| -0.00035 | 0.01017 |
| -0.00038 | 0.010213 |
| -0.00041 | 0.010257 |
| -0.00045 | 0.0103 |
| -0.00048 | 0.010344 |
| -0.00051 | 0.010388 |
| -0.00055 | 0.010432 |
| -0.00058 | 0.010477 |
| -0.00061 | 0.010521 |
| -0.00065 | 0.010566 |
| -0.00068 | 0.010612 |
| -0.00072 | 0.010658 |
| -0.00075 | 0.010703 |
| -0.00079 | 0.01075 |
| -0.00082 | 0.010796 |
| -0.00086 | 0.010843 |
| -0.0009 | 0.01089 |
| -0.00093 | 0.010938 |

| | |
|---|---|
| -0.00097 | 0.010985 |
| -0.00101 | 0.011033 |
| -0.00105 | 0.011082 |
| -0.00109 | 0.011131 |
| -0.00112 | 0.01118 |
| -0.00116 | 0.011229 |
| -0.0012 | 0.011278 |
| -0.00124 | 0.011328 |
| -0.00128 | 0.011379 |
| -0.00132 | 0.011429 |
| -0.00136 | 0.01148 |
| -0.0014 | 0.011532 |
| -0.00145 | 0.011583 |
| -0.00149 | 0.011635 |
| -0.00153 | 0.011688 |
| -0.00157 | 0.011741 |
| -0.00162 | 0.011794 |
| -0.00166 | 0.011847 |
| -0.0017 | 0.011901 |
| -0.00175 | 0.011955 |
| -0.00179 | 0.01201 |
| -0.00184 | 0.012065 |
| -0.00188 | 0.01212 |
| -0.00193 | 0.012176 |
| -0.00198 | 0.012232 |
| -0.00202 | 0.012289 |
| -0.00207 | 0.012346 |
| -0.00212 | 0.012403 |
| -0.00217 | 0.012461 |
| -0.00222 | 0.012519 |
| -0.00227 | 0.012578 |
| -0.00232 | 0.012637 |
| -0.00237 | 0.012697 |
| -0.00242 | 0.012757 |
| -0.00247 | 0.012817 |
| -0.00252 | 0.012878 |
| -0.00257 | 0.012939 |
| -0.00262 | 0.013001 |
| -0.00268 | 0.013064 |
| -0.00273 | 0.013126 |
| -0.00279 | 0.01319 |
| -0.00284 | 0.013253 |
| -0.0029 | 0.013317 |
| -0.00295 | 0.013382 |
| -0.00301 | 0.013447 |
| -0.00307 | 0.013513 |
| -0.00313 | 0.013579 |
| -0.00318 | 0.013646 |
| -0.00324 | 0.013714 |

| | |
|---|---|
| -0.0033 | 0.013781 |
| -0.00336 | 0.01385 |
| -0.00342 | 0.013919 |
| -0.00349 | 0.013988 |
| -0.00355 | 0.014058 |
| -0.00361 | 0.014129 |
| -0.00367 | 0.0142 |
| -0.00374 | 0.014272 |
| -0.0038 | 0.014345 |
| -0.00387 | 0.014418 |
| -0.00394 | 0.014491 |
| -0.004 | 0.014565 |
| -0.00407 | 0.01464 |
| -0.00414 | 0.014716 |
| -0.00421 | 0.014792 |
| -0.00428 | 0.014869 |
| -0.00435 | 0.014946 |
| -0.00442 | 0.015024 |
| -0.00449 | 0.015103 |
| -0.00457 | 0.015183 |
| -0.00464 | 0.015263 |
| -0.00472 | 0.015344 |
| -0.00479 | 0.015426 |
| -0.00487 | 0.015508 |
| -0.00495 | 0.015591 |
| -0.00502 | 0.015675 |
| -0.0051 | 0.01576 |
| -0.00518 | 0.015845 |
| -0.00527 | 0.015931 |
| -0.00535 | 0.016018 |
| -0.00543 | 0.016106 |
| -0.00551 | 0.016195 |
| -0.0056 | 0.016284 |
| -0.00569 | 0.016374 |
| -0.00577 | 0.016465 |
| -0.00586 | 0.016557 |
| -0.00595 | 0.01665 |
| -0.00604 | 0.016744 |
| -0.00613 | 0.016839 |
| -0.00622 | 0.016934 |
| -0.00632 | 0.017031 |
| -0.00641 | 0.017128 |
| -0.00651 | 0.017226 |
| -0.0066 | 0.017326 |
| -0.0067 | 0.017426 |
| -0.0068 | 0.017527 |
| -0.0069 | 0.01763 |
| -0.007 | 0.017733 |
| -0.00711 | 0.017838 |

| | |
|---|---|
| -0.00721 | 0.017943 |
| -0.00732 | 0.01805 |
| -0.00743 | 0.018157 |
| -0.00753 | 0.018266 |
| -0.00764 | 0.018376 |
| -0.00776 | 0.018487 |
| -0.00787 | 0.018599 |
| -0.00798 | 0.018713 |
| -0.0081 | 0.018827 |
| -0.00822 | 0.018943 |
| -0.00833 | 0.01906 |
| -0.00845 | 0.019179 |
| -0.00858 | 0.019298 |
| -0.0087 | 0.019419 |
| -0.00883 | 0.019542 |
| -0.00895 | 0.019665 |
| -0.00908 | 0.019791 |
| -0.00921 | 0.019917 |
| -0.00934 | 0.020045 |
| -0.00948 | 0.020174 |
| -0.00961 | 0.020305 |
| -0.00975 | 0.020437 |
| -0.00989 | 0.020571 |
| -0.01003 | 0.020706 |
| -0.01018 | 0.020843 |
| -0.01032 | 0.020982 |
| -0.01047 | 0.021122 |
| -0.01062 | 0.021264 |
| -0.01077 | 0.021407 |
| -0.01093 | 0.021553 |
| -0.01108 | 0.0217 |
| -0.01124 | 0.021848 |
| -0.0114 | 0.021999 |
| -0.01157 | 0.022152 |
| -0.01173 | 0.022306 |
| -0.0119 | 0.022462 |
| -0.01207 | 0.02262 |
| -0.01224 | 0.022781 |
| -0.01242 | 0.022943 |
| -0.0126 | 0.023107 |
| -0.01278 | 0.023274 |
| -0.01297 | 0.023442 |
| -0.01315 | 0.023613 |
| -0.01334 | 0.023786 |
| -0.01354 | 0.023961 |
| -0.01373 | 0.024139 |
| -0.01393 | 0.024319 |
| -0.01413 | 0.024501 |
| -0.01434 | 0.024686 |

| | |
|---|---|
| -0.01455 | 0.024874 |
| -0.01476 | 0.025064 |
| -0.01498 | 0.025256 |
| -0.01519 | 0.025452 |
| -0.01542 | 0.02565 |
| -0.01564 | 0.02585 |
| -0.01587 | 0.026054 |
| -0.01611 | 0.02626 |
| -0.01635 | 0.02647 |
| -0.01659 | 0.026682 |
| -0.01683 | 0.026898 |
| -0.01708 | 0.027117 |
| -0.01734 | 0.027339 |
| -0.0176 | 0.027564 |
| -0.01786 | 0.027793 |
| -0.01813 | 0.028025 |
| -0.0184 | 0.028261 |
| -0.01868 | 0.0285 |
| -0.01896 | 0.028743 |
| -0.01925 | 0.028989 |
| -0.01954 | 0.02924 |
| -0.01984 | 0.029494 |
| -0.02015 | 0.029753 |
| -0.02046 | 0.030016 |
| -0.02077 | 0.030283 |
| -0.02109 | 0.030554 |
| -0.02142 | 0.030829 |
| -0.02175 | 0.03111 |
| -0.02209 | 0.031394 |
| -0.02244 | 0.031684 |
| -0.02279 | 0.031979 |
| -0.02315 | 0.032278 |
| -0.02352 | 0.032583 |
| -0.02389 | 0.032892 |
| -0.02427 | 0.033208 |
| -0.02466 | 0.033529 |
| -0.02506 | 0.033855 |
| -0.02546 | 0.034187 |
| -0.02588 | 0.034525 |
| -0.0263 | 0.03487 |
| -0.02673 | 0.03522 |
| -0.02717 | 0.035578 |
| -0.02762 | 0.035941 |
| -0.02808 | 0.036312 |
| -0.02855 | 0.036689 |
| -0.02902 | 0.037074 |
| -0.02951 | 0.037466 |
| -0.03001 | 0.037866 |
| -0.03052 | 0.038273 |

| | |
|---|---|
| -0.03104 | 0.038689 |
| -0.03158 | 0.039113 |
| -0.03212 | 0.039545 |
| -0.03268 | 0.039986 |
| -0.03325 | 0.040436 |
| -0.03384 | 0.040896 |
| -0.03443 | 0.041365 |
| -0.03504 | 0.041843 |
| -0.03567 | 0.042332 |
| -0.03631 | 0.042832 |
| -0.03697 | 0.043342 |
| -0.03764 | 0.043863 |
| -0.03833 | 0.044396 |
| -0.03904 | 0.044941 |
| -0.03976 | 0.045498 |
| -0.0405 | 0.046067 |
| -0.04126 | 0.04665 |
| -0.04204 | 0.047246 |
| -0.04284 | 0.047856 |
| -0.04367 | 0.048481 |
| -0.04451 | 0.04912 |
| -0.04538 | 0.049775 |
| -0.04627 | 0.050446 |
| -0.04718 | 0.051133 |
| -0.04812 | 0.051837 |
| -0.04908 | 0.05256 |
| -0.05007 | 0.0533 |
| -0.05109 | 0.05406 |
| -0.05214 | 0.054839 |
| -0.05322 | 0.055639 |
| -0.05433 | 0.05646 |
| -0.05548 | 0.057303 |
| -0.05665 | 0.05817 |
| -0.05787 | 0.05906 |
| -0.05912 | 0.059975 |
| -0.06041 | 0.060916 |
| -0.06174 | 0.061884 |
| -0.06311 | 0.062879 |
| -0.06452 | 0.063904 |
| -0.06598 | 0.064959 |
| -0.06749 | 0.066046 |
| -0.06905 | 0.067166 |
| -0.07066 | 0.068321 |
| -0.07232 | 0.069512 |
| -0.07404 | 0.07074 |
| -0.07582 | 0.072007 |
| -0.07767 | 0.073316 |
| -0.07958 | 0.074668 |
| -0.08155 | 0.076065 |

| | |
|---|---|
| -0.0836 | 0.07751 |
| -0.08573 | 0.079004 |
| -0.08794 | 0.08055 |
| -0.09023 | 0.082151 |
| -0.0926 | 0.08381 |
| -0.09507 | 0.085529 |
| -0.09764 | 0.087311 |
| -0.10032 | 0.089161 |
| -0.1031 | 0.091081 |
| -0.106 | 0.093076 |
| -0.10901 | 0.09515 |
| -0.11216 | 0.097306 |
| -0.11545 | 0.099551 |
| -0.11888 | 0.101888 |
| -0.12246 | 0.104324 |
| -0.12621 | 0.106865 |
| -0.13013 | 0.109517 |
| -0.13424 | 0.112286 |
| -0.13854 | 0.115181 |
| -0.14305 | 0.11821 |
| -0.14779 | 0.121381 |
| -0.15277 | 0.124704 |
| -0.15801 | 0.12819 |
| -0.16352 | 0.131849 |
| -0.16932 | 0.135695 |
| -0.17545 | 0.139741 |
| -0.18191 | 0.144002 |
| -0.18875 | 0.148494 |
| -0.19598 | 0.153235 |
| -0.20365 | 0.158245 |
| -0.21178 | 0.163546 |
| -0.22041 | 0.169162 |
| -0.2296 | 0.17512 |
| -0.23938 | 0.18145 |
| -0.24982 | 0.188185 |
| -0.26098 | 0.195362 |
| -0.27291 | 0.203023 |
| -0.28571 | 0.211216 |
| -0.29946 | 0.219992 |
| -0.31425 | 0.229411 |
| -0.33021 | 0.239542 |
| -0.34745 | 0.250462 |
| -0.36612 | 0.262259 |
| -0.3864 | 0.275033 |
| -0.40847 | 0.288903 |
| -0.43257 | 0.304004 |
| -0.45895 | 0.320492 |
| -0.48793 | 0.338553 |
| -0.51986 | 0.358402 |

| | |
|---|---|
| -0.55517 | 0.380296 |
| -0.59438 | 0.404539 |
| -0.6381 | 0.431499 |
| -0.68707 | 0.461615 |
| -0.74219 | 0.495427 |
| -0.80459 | 0.533594 |
| -0.87563 | 0.576936 |
| -0.95706 | 0.626478 |
| -1.05107 | 0.683523 |
| -1.1605 | 0.74974 |
| -1.28903 | 0.827308 |
| -1.44157 | 0.919109 |
| -1.6247 | 1.02903 |
| -1.84754 | 1.16242 |
| -2.12294 | 1.32682 |
| -2.46954 | 1.53316 |
| -2.91529 | 1.7978 |
| -3.50376 | 2.14621 |
| -4.30614 | 2.61997 |
| -5.44583 | 3.29105 |
| -7.1535 | 4.29378 |
| -9.90568 | 5.90539 |
| -14.8354 | 8.78411 |
| -25.2768 | 14.8642 |
| -55.4647 | 32.393 |
| -256.649 | 148.86 |

**Table S1-A2.** Keypoints used for generating an hyperbolic notch with opening angle $2\alpha$ = 45 degrees and radius of the notch tip equal to 0.01mm.

| X | Y |
|---|---|
| 0.004286 | 0 |
| 0.004286 | 1.29E-05 |
| 0.004286 | 2.57E-05 |
| 0.004286 | 3.86E-05 |
| 0.004286 | 5.14E-05 |
| 0.004286 | 6.43E-05 |
| 0.004285 | 7.71E-05 |
| 0.004285 | 9E-05 |
| 0.004285 | 0.000103 |
| 0.004285 | 0.000116 |
| 0.004285 | 0.000129 |
| 0.004285 | 0.000141 |
| 0.004285 | 0.000154 |
| 0.004284 | 0.000167 |
| 0.004284 | 0.00018 |
| 0.004284 | 0.000193 |
| 0.004284 | 0.000206 |
| 0.004283 | 0.000219 |

| | |
|---|---|
| 0.004283 | 0.000232 |
| 0.004283 | 0.000244 |
| 0.004282 | 0.000257 |
| 0.004282 | 0.00027 |
| 0.004282 | 0.000283 |
| 0.004281 | 0.000296 |
| 0.004281 | 0.000309 |
| 0.004281 | 0.000322 |
| 0.00428 | 0.000335 |
| 0.00428 | 0.000347 |
| 0.004279 | 0.00036 |
| 0.004279 | 0.000373 |
| 0.004278 | 0.000386 |
| 0.004278 | 0.000399 |
| 0.004277 | 0.000412 |
| 0.004277 | 0.000425 |
| 0.004276 | 0.000438 |
| 0.004276 | 0.000451 |
| 0.004275 | 0.000464 |
| 0.004274 | 0.000476 |
| 0.004274 | 0.000489 |
| 0.004273 | 0.000502 |
| 0.004272 | 0.000515 |
| 0.004272 | 0.000528 |
| 0.004271 | 0.000541 |
| 0.00427 | 0.000554 |
| 0.00427 | 0.000567 |
| 0.004269 | 0.00058 |
| 0.004268 | 0.000593 |
| 0.004267 | 0.000606 |
| 0.004267 | 0.000619 |
| 0.004266 | 0.000632 |
| 0.004265 | 0.000645 |
| 0.004264 | 0.000658 |
| 0.004263 | 0.000671 |
| 0.004262 | 0.000683 |
| 0.004261 | 0.000696 |
| 0.004261 | 0.000709 |
| 0.00426 | 0.000722 |
| 0.004259 | 0.000735 |
| 0.004258 | 0.000748 |
| 0.004257 | 0.000761 |
| 0.004256 | 0.000774 |
| 0.004255 | 0.000787 |
| 0.004254 | 0.0008 |
| 0.004253 | 0.000813 |
| 0.004252 | 0.000826 |
| 0.004251 | 0.00084 |
| 0.004249 | 0.000853 |

| | |
|---|---|
| 0.004248 | 0.000866 |
| 0.004247 | 0.000879 |
| 0.004246 | 0.000892 |
| 0.004245 | 0.000905 |
| 0.004244 | 0.000918 |
| 0.004242 | 0.000931 |
| 0.004241 | 0.000944 |
| 0.00424 | 0.000957 |
| 0.004239 | 0.00097 |
| 0.004237 | 0.000983 |
| 0.004236 | 0.000996 |
| 0.004235 | 0.001009 |
| 0.004234 | 0.001023 |
| 0.004232 | 0.001036 |
| 0.004231 | 0.001049 |
| 0.004229 | 0.001062 |
| 0.004228 | 0.001075 |
| 0.004227 | 0.001088 |
| 0.004225 | 0.001101 |
| 0.004224 | 0.001115 |
| 0.004222 | 0.001128 |
| 0.004221 | 0.001141 |
| 0.004219 | 0.001154 |
| 0.004218 | 0.001167 |
| 0.004216 | 0.00118 |
| 0.004215 | 0.001194 |
| 0.004213 | 0.001207 |
| 0.004211 | 0.00122 |
| 0.00421 | 0.001233 |
| 0.004208 | 0.001247 |
| 0.004207 | 0.00126 |
| 0.004205 | 0.001273 |
| 0.004203 | 0.001286 |
| 0.004201 | 0.0013 |
| 0.0042 | 0.001313 |
| 0.004198 | 0.001326 |
| 0.004196 | 0.00134 |
| 0.004194 | 0.001353 |
| 0.004193 | 0.001366 |
| 0.004191 | 0.00138 |
| 0.004189 | 0.001393 |
| 0.004187 | 0.001406 |
| 0.004185 | 0.00142 |
| 0.004183 | 0.001433 |
| 0.004181 | 0.001446 |
| 0.00418 | 0.00146 |
| 0.004178 | 0.001473 |
| 0.004176 | 0.001486 |
| 0.004174 | 0.0015 |

| | |
|---|---|
| 0.004172 | 0.001513 |
| 0.00417 | 0.001527 |
| 0.004168 | 0.00154 |
| 0.004165 | 0.001554 |
| 0.004163 | 0.001567 |
| 0.004161 | 0.001581 |
| 0.004159 | 0.001594 |
| 0.004157 | 0.001608 |
| 0.004155 | 0.001621 |
| 0.004153 | 0.001635 |
| 0.00415 | 0.001648 |
| 0.004148 | 0.001662 |
| 0.004146 | 0.001675 |
| 0.004144 | 0.001689 |
| 0.004141 | 0.001702 |
| 0.004139 | 0.001716 |
| 0.004137 | 0.00173 |
| 0.004135 | 0.001743 |
| 0.004132 | 0.001757 |
| 0.00413 | 0.00177 |
| 0.004127 | 0.001784 |
| 0.004125 | 0.001798 |
| 0.004122 | 0.001811 |
| 0.00412 | 0.001825 |
| 0.004118 | 0.001839 |
| 0.004115 | 0.001852 |
| 0.004113 | 0.001866 |
| 0.00411 | 0.00188 |
| 0.004107 | 0.001894 |
| 0.004105 | 0.001907 |
| 0.004102 | 0.001921 |
| 0.0041 | 0.001935 |
| 0.004097 | 0.001949 |
| 0.004094 | 0.001963 |
| 0.004092 | 0.001976 |
| 0.004089 | 0.00199 |
| 0.004086 | 0.002004 |
| 0.004083 | 0.002018 |
| 0.004081 | 0.002032 |
| 0.004078 | 0.002046 |
| 0.004075 | 0.00206 |
| 0.004072 | 0.002074 |
| 0.004069 | 0.002088 |
| 0.004066 | 0.002102 |
| 0.004063 | 0.002116 |
| 0.004061 | 0.002129 |
| 0.004058 | 0.002143 |
| 0.004055 | 0.002157 |
| 0.004052 | 0.002172 |

| | |
|---|---|
| 0.004049 | 0.002186 |
| 0.004046 | 0.0022 |
| 0.004043 | 0.002214 |
| 0.004039 | 0.002228 |
| 0.004036 | 0.002242 |
| 0.004033 | 0.002256 |
| 0.00403 | 0.00227 |
| 0.004027 | 0.002284 |
| 0.004024 | 0.002298 |
| 0.00402 | 0.002313 |
| 0.004017 | 0.002327 |
| 0.004014 | 0.002341 |
| 0.004011 | 0.002355 |
| 0.004007 | 0.00237 |
| 0.004004 | 0.002384 |
| 0.004001 | 0.002398 |
| 0.003997 | 0.002412 |
| 0.003994 | 0.002427 |
| 0.00399 | 0.002441 |
| 0.003987 | 0.002455 |
| 0.003984 | 0.00247 |
| 0.00398 | 0.002484 |
| 0.003977 | 0.002499 |
| 0.003973 | 0.002513 |
| 0.003969 | 0.002528 |
| 0.003966 | 0.002542 |
| 0.003962 | 0.002556 |
| 0.003959 | 0.002571 |
| 0.003955 | 0.002585 |
| 0.003951 | 0.0026 |
| 0.003947 | 0.002615 |
| 0.003944 | 0.002629 |
| 0.00394 | 0.002644 |
| 0.003936 | 0.002658 |
| 0.003932 | 0.002673 |
| 0.003929 | 0.002688 |
| 0.003925 | 0.002702 |
| 0.003921 | 0.002717 |
| 0.003917 | 0.002732 |
| 0.003913 | 0.002746 |
| 0.003909 | 0.002761 |
| 0.003905 | 0.002776 |
| 0.003901 | 0.002791 |
| 0.003897 | 0.002806 |
| 0.003893 | 0.00282 |
| 0.003889 | 0.002835 |
| 0.003885 | 0.00285 |
| 0.00388 | 0.002865 |
| 0.003876 | 0.00288 |

| | |
|---|---|
| 0.003872 | 0.002895 |
| 0.003868 | 0.00291 |
| 0.003864 | 0.002925 |
| 0.003859 | 0.00294 |
| 0.003855 | 0.002955 |
| 0.003851 | 0.00297 |
| 0.003846 | 0.002985 |
| 0.003842 | 0.003 |
| 0.003837 | 0.003015 |
| 0.003833 | 0.00303 |
| 0.003828 | 0.003046 |
| 0.003824 | 0.003061 |
| 0.003819 | 0.003076 |
| 0.003815 | 0.003091 |
| 0.00381 | 0.003106 |
| 0.003806 | 0.003122 |
| 0.003801 | 0.003137 |
| 0.003796 | 0.003152 |
| 0.003792 | 0.003168 |
| 0.003787 | 0.003183 |
| 0.003782 | 0.003199 |
| 0.003777 | 0.003214 |
| 0.003772 | 0.003229 |
| 0.003768 | 0.003245 |
| 0.003763 | 0.00326 |
| 0.003758 | 0.003276 |
| 0.003753 | 0.003291 |
| 0.003748 | 0.003307 |
| 0.003743 | 0.003323 |
| 0.003738 | 0.003338 |
| 0.003733 | 0.003354 |
| 0.003728 | 0.00337 |
| 0.003722 | 0.003385 |
| 0.003717 | 0.003401 |
| 0.003712 | 0.003417 |
| 0.003707 | 0.003433 |
| 0.003702 | 0.003448 |
| 0.003696 | 0.003464 |
| 0.003691 | 0.00348 |
| 0.003686 | 0.003496 |
| 0.00368 | 0.003512 |
| 0.003675 | 0.003528 |
| 0.003669 | 0.003544 |
| 0.003664 | 0.00356 |
| 0.003658 | 0.003576 |
| 0.003653 | 0.003592 |
| 0.003647 | 0.003608 |
| 0.003642 | 0.003624 |
| 0.003636 | 0.00364 |

| | |
|---|---|
| 0.00363 | 0.003657 |
| 0.003625 | 0.003673 |
| 0.003619 | 0.003689 |
| 0.003613 | 0.003705 |
| 0.003607 | 0.003722 |
| 0.003601 | 0.003738 |
| 0.003596 | 0.003754 |
| 0.00359 | 0.003771 |
| 0.003584 | 0.003787 |
| 0.003578 | 0.003804 |
| 0.003572 | 0.00382 |
| 0.003566 | 0.003837 |
| 0.00356 | 0.003853 |
| 0.003553 | 0.00387 |
| 0.003547 | 0.003886 |
| 0.003541 | 0.003903 |
| 0.003535 | 0.00392 |
| 0.003528 | 0.003936 |
| 0.003522 | 0.003953 |
| 0.003516 | 0.00397 |
| 0.003509 | 0.003987 |
| 0.003503 | 0.004004 |
| 0.003497 | 0.004021 |
| 0.00349 | 0.004037 |
| 0.003483 | 0.004054 |
| 0.003477 | 0.004071 |
| 0.00347 | 0.004088 |
| 0.003464 | 0.004106 |
| 0.003457 | 0.004123 |
| 0.00345 | 0.00414 |
| 0.003443 | 0.004157 |
| 0.003437 | 0.004174 |
| 0.00343 | 0.004191 |
| 0.003423 | 0.004209 |
| 0.003416 | 0.004226 |
| 0.003409 | 0.004243 |
| 0.003402 | 0.004261 |
| 0.003395 | 0.004278 |
| 0.003388 | 0.004296 |
| 0.003381 | 0.004313 |
| 0.003373 | 0.004331 |
| 0.003366 | 0.004348 |
| 0.003359 | 0.004366 |
| 0.003352 | 0.004383 |
| 0.003344 | 0.004401 |
| 0.003337 | 0.004419 |
| 0.003329 | 0.004437 |
| 0.003322 | 0.004454 |
| 0.003314 | 0.004472 |

| | |
|---|---|
| 0.003307 | 0.00449 |
| 0.003299 | 0.004508 |
| 0.003292 | 0.004526 |
| 0.003284 | 0.004544 |
| 0.003276 | 0.004562 |
| 0.003268 | 0.00458 |
| 0.00326 | 0.004598 |
| 0.003253 | 0.004616 |
| 0.003245 | 0.004635 |
| 0.003237 | 0.004653 |
| 0.003229 | 0.004671 |
| 0.003221 | 0.004689 |
| 0.003212 | 0.004708 |
| 0.003204 | 0.004726 |
| 0.003196 | 0.004745 |
| 0.003188 | 0.004763 |
| 0.00318 | 0.004782 |
| 0.003171 | 0.0048 |
| 0.003163 | 0.004819 |
| 0.003154 | 0.004838 |
| 0.003146 | 0.004856 |
| 0.003137 | 0.004875 |
| 0.003129 | 0.004894 |
| 0.00312 | 0.004913 |
| 0.003111 | 0.004932 |
| 0.003103 | 0.004951 |
| 0.003094 | 0.00497 |
| 0.003085 | 0.004989 |
| 0.003076 | 0.005008 |
| 0.003067 | 0.005027 |
| 0.003058 | 0.005046 |
| 0.003049 | 0.005066 |
| 0.00304 | 0.005085 |
| 0.003031 | 0.005104 |
| 0.003021 | 0.005124 |
| 0.003012 | 0.005143 |
| 0.003003 | 0.005163 |
| 0.002993 | 0.005182 |
| 0.002984 | 0.005202 |
| 0.002974 | 0.005221 |
| 0.002965 | 0.005241 |
| 0.002955 | 0.005261 |
| 0.002945 | 0.005281 |
| 0.002936 | 0.005301 |
| 0.002926 | 0.00532 |
| 0.002916 | 0.00534 |
| 0.002906 | 0.00536 |
| 0.002896 | 0.005381 |
| 0.002886 | 0.005401 |

| | |
|---|---|
| 0.002876 | 0.005421 |
| 0.002866 | 0.005441 |
| 0.002856 | 0.005461 |
| 0.002845 | 0.005482 |
| 0.002835 | 0.005502 |
| 0.002825 | 0.005523 |
| 0.002814 | 0.005543 |
| 0.002804 | 0.005564 |
| 0.002793 | 0.005584 |
| 0.002783 | 0.005605 |
| 0.002772 | 0.005626 |
| 0.002761 | 0.005647 |
| 0.00275 | 0.005667 |
| 0.002739 | 0.005688 |
| 0.002728 | 0.005709 |
| 0.002717 | 0.00573 |
| 0.002706 | 0.005751 |
| 0.002695 | 0.005773 |
| 0.002684 | 0.005794 |
| 0.002673 | 0.005815 |
| 0.002661 | 0.005837 |
| 0.00265 | 0.005858 |
| 0.002638 | 0.005879 |
| 0.002627 | 0.005901 |
| 0.002615 | 0.005923 |
| 0.002603 | 0.005944 |
| 0.002592 | 0.005966 |
| 0.00258 | 0.005988 |
| 0.002568 | 0.00601 |
| 0.002556 | 0.006032 |
| 0.002544 | 0.006054 |
| 0.002532 | 0.006076 |
| 0.002519 | 0.006098 |
| 0.002507 | 0.00612 |
| 0.002495 | 0.006142 |
| 0.002482 | 0.006164 |
| 0.00247 | 0.006187 |
| 0.002457 | 0.006209 |
| 0.002444 | 0.006232 |
| 0.002432 | 0.006255 |
| 0.002419 | 0.006277 |
| 0.002406 | 0.0063 |
| 0.002393 | 0.006323 |
| 0.00238 | 0.006346 |
| 0.002367 | 0.006369 |
| 0.002353 | 0.006392 |
| 0.00234 | 0.006415 |
| 0.002327 | 0.006438 |
| 0.002313 | 0.006461 |

| | |
|---|---|
| 0.0023 | 0.006485 |
| 0.002286 | 0.006508 |
| 0.002272 | 0.006532 |
| 0.002258 | 0.006555 |
| 0.002245 | 0.006579 |
| 0.002231 | 0.006602 |
| 0.002216 | 0.006626 |
| 0.002202 | 0.00665 |
| 0.002188 | 0.006674 |
| 0.002174 | 0.006698 |
| 0.002159 | 0.006722 |
| 0.002145 | 0.006746 |
| 0.00213 | 0.006771 |
| 0.002115 | 0.006795 |
| 0.002101 | 0.00682 |
| 0.002086 | 0.006844 |
| 0.002071 | 0.006869 |
| 0.002056 | 0.006893 |
| 0.00204 | 0.006918 |
| 0.002025 | 0.006943 |
| 0.00201 | 0.006968 |
| 0.001994 | 0.006993 |
| 0.001979 | 0.007018 |
| 0.001963 | 0.007044 |
| 0.001947 | 0.007069 |
| 0.001931 | 0.007094 |
| 0.001915 | 0.00712 |
| 0.001899 | 0.007145 |
| 0.001883 | 0.007171 |
| 0.001867 | 0.007197 |
| 0.00185 | 0.007223 |
| 0.001834 | 0.007249 |
| 0.001817 | 0.007275 |
| 0.001801 | 0.007301 |
| 0.001784 | 0.007327 |
| 0.001767 | 0.007353 |
| 0.00175 | 0.00738 |
| 0.001733 | 0.007406 |
| 0.001715 | 0.007433 |
| 0.001698 | 0.00746 |
| 0.00168 | 0.007487 |
| 0.001663 | 0.007513 |
| 0.001645 | 0.007541 |
| 0.001627 | 0.007568 |
| 0.001609 | 0.007595 |
| 0.001591 | 0.007622 |
| 0.001573 | 0.00765 |
| 0.001555 | 0.007677 |
| 0.001536 | 0.007705 |

| | |
|---|---|
| 0.001518 | 0.007733 |
| 0.001499 | 0.007761 |
| 0.00148 | 0.007789 |
| 0.001461 | 0.007817 |
| 0.001442 | 0.007845 |
| 0.001423 | 0.007873 |
| 0.001404 | 0.007902 |
| 0.001384 | 0.00793 |
| 0.001365 | 0.007959 |
| 0.001345 | 0.007987 |
| 0.001325 | 0.008016 |
| 0.001305 | 0.008045 |
| 0.001285 | 0.008074 |
| 0.001265 | 0.008104 |
| 0.001244 | 0.008133 |
| 0.001224 | 0.008162 |
| 0.001203 | 0.008192 |
| 0.001182 | 0.008222 |
| 0.001161 | 0.008252 |
| 0.00114 | 0.008282 |
| 0.001119 | 0.008312 |
| 0.001097 | 0.008342 |
| 0.001076 | 0.008372 |
| 0.001054 | 0.008403 |
| 0.001032 | 0.008433 |
| 0.00101 | 0.008464 |
| 0.000988 | 0.008495 |
| 0.000966 | 0.008526 |
| 0.000943 | 0.008557 |
| 0.000921 | 0.008588 |
| 0.000898 | 0.008619 |
| 0.000875 | 0.008651 |
| 0.000852 | 0.008683 |
| 0.000829 | 0.008714 |
| 0.000805 | 0.008746 |
| 0.000782 | 0.008778 |
| 0.000758 | 0.008811 |
| 0.000734 | 0.008843 |
| 0.00071 | 0.008875 |
| 0.000685 | 0.008908 |
| 0.000661 | 0.008941 |
| 0.000636 | 0.008974 |
| 0.000612 | 0.009007 |
| 0.000587 | 0.00904 |
| 0.000561 | 0.009073 |
| 0.000536 | 0.009107 |
| 0.000511 | 0.009141 |
| 0.000485 | 0.009174 |
| 0.000459 | 0.009208 |

| | |
|---|---|
| 0.000433 | 0.009243 |
| 0.000407 | 0.009277 |
| 0.00038 | 0.009311 |
| 0.000353 | 0.009346 |
| 0.000327 | 0.009381 |
| 0.000299 | 0.009416 |
| 0.000272 | 0.009451 |
| 0.000245 | 0.009486 |
| 0.000217 | 0.009522 |
| 0.000189 | 0.009557 |
| 0.000161 | 0.009593 |
| 0.000133 | 0.009629 |
| 0.000104 | 0.009665 |
| 7.56E-05 | 0.009701 |
| 4.67E-05 | 0.009738 |
| 1.76E-05 | 0.009774 |
| -1.2E-05 | 0.009811 |
| -4.1E-05 | 0.009848 |
| -7.1E-05 | 0.009886 |
| -0.0001 | 0.009923 |
| -0.00013 | 0.00996 |
| -0.00016 | 0.009998 |
| -0.00019 | 0.010036 |
| -0.00022 | 0.010074 |
| -0.00025 | 0.010113 |
| -0.00029 | 0.010151 |
| -0.00032 | 0.01019 |
| -0.00035 | 0.010229 |
| -0.00038 | 0.010268 |
| -0.00041 | 0.010307 |
| -0.00045 | 0.010347 |
| -0.00048 | 0.010386 |
| -0.00051 | 0.010426 |
| -0.00055 | 0.010467 |
| -0.00058 | 0.010507 |
| -0.00061 | 0.010547 |
| -0.00065 | 0.010588 |
| -0.00068 | 0.010629 |
| -0.00072 | 0.01067 |
| -0.00075 | 0.010712 |
| -0.00079 | 0.010753 |
| -0.00082 | 0.010795 |
| -0.00086 | 0.010837 |
| -0.0009 | 0.01088 |
| -0.00093 | 0.010922 |
| -0.00097 | 0.010965 |
| -0.00101 | 0.011008 |
| -0.00104 | 0.011051 |
| -0.00108 | 0.011095 |

| | |
|---|---|
| -0.00112 | 0.011139 |
| -0.00116 | 0.011183 |
| -0.0012 | 0.011227 |
| -0.00124 | 0.011271 |
| -0.00128 | 0.011316 |
| -0.00131 | 0.011361 |
| -0.00135 | 0.011406 |
| -0.00139 | 0.011452 |
| -0.00144 | 0.011497 |
| -0.00148 | 0.011543 |
| -0.00152 | 0.01159 |
| -0.00156 | 0.011636 |
| -0.0016 | 0.011683 |
| -0.00164 | 0.01173 |
| -0.00169 | 0.011777 |
| -0.00173 | 0.011825 |
| -0.00177 | 0.011873 |
| -0.00182 | 0.011921 |
| -0.00186 | 0.01197 |
| -0.00191 | 0.012018 |
| -0.00195 | 0.012067 |
| -0.002 | 0.012117 |
| -0.00204 | 0.012166 |
| -0.00209 | 0.012216 |
| -0.00213 | 0.012267 |
| -0.00218 | 0.012317 |
| -0.00223 | 0.012368 |
| -0.00228 | 0.012419 |
| -0.00232 | 0.012471 |
| -0.00237 | 0.012523 |
| -0.00242 | 0.012575 |
| -0.00247 | 0.012627 |
| -0.00252 | 0.01268 |
| -0.00257 | 0.012733 |
| -0.00262 | 0.012787 |
| -0.00267 | 0.01284 |
| -0.00272 | 0.012895 |
| -0.00278 | 0.012949 |
| -0.00283 | 0.013004 |
| -0.00288 | 0.013059 |
| -0.00294 | 0.013115 |
| -0.00299 | 0.013171 |
| -0.00304 | 0.013227 |
| -0.0031 | 0.013284 |
| -0.00315 | 0.013341 |
| -0.00321 | 0.013398 |
| -0.00327 | 0.013456 |
| -0.00332 | 0.013514 |
| -0.00338 | 0.013573 |

| | |
|---|---|
| -0.00344 | 0.013632 |
| -0.0035 | 0.013691 |
| -0.00356 | 0.013751 |
| -0.00362 | 0.013811 |
| -0.00368 | 0.013871 |
| -0.00374 | 0.013932 |
| -0.0038 | 0.013994 |
| -0.00386 | 0.014056 |
| -0.00393 | 0.014118 |
| -0.00399 | 0.014181 |
| -0.00405 | 0.014244 |
| -0.00412 | 0.014308 |
| -0.00418 | 0.014372 |
| -0.00425 | 0.014436 |
| -0.00431 | 0.014501 |
| -0.00438 | 0.014567 |
| -0.00445 | 0.014632 |
| -0.00452 | 0.014699 |
| -0.00459 | 0.014766 |
| -0.00466 | 0.014833 |
| -0.00473 | 0.014901 |
| -0.0048 | 0.014969 |
| -0.00487 | 0.015038 |
| -0.00494 | 0.015107 |
| -0.00502 | 0.015177 |
| -0.00509 | 0.015248 |
| -0.00517 | 0.015319 |
| -0.00524 | 0.01539 |
| -0.00532 | 0.015462 |
| -0.00539 | 0.015535 |
| -0.00547 | 0.015608 |
| -0.00555 | 0.015682 |
| -0.00563 | 0.015756 |
| -0.00571 | 0.015831 |
| -0.00579 | 0.015906 |
| -0.00587 | 0.015982 |
| -0.00596 | 0.016059 |
| -0.00604 | 0.016136 |
| -0.00613 | 0.016214 |
| -0.00621 | 0.016292 |
| -0.0063 | 0.016371 |
| -0.00638 | 0.016451 |
| -0.00647 | 0.016532 |
| -0.00656 | 0.016613 |
| -0.00665 | 0.016694 |
| -0.00674 | 0.016777 |
| -0.00684 | 0.01686 |
| -0.00693 | 0.016943 |
| -0.00702 | 0.017028 |

| | |
|---|---|
| -0.00712 | 0.017113 |
| -0.00721 | 0.017199 |
| -0.00731 | 0.017285 |
| -0.00741 | 0.017373 |
| -0.00751 | 0.017461 |
| -0.00761 | 0.017549 |
| -0.00771 | 0.017639 |
| -0.00782 | 0.017729 |
| -0.00792 | 0.01782 |
| -0.00803 | 0.017912 |
| -0.00813 | 0.018005 |
| -0.00824 | 0.018099 |
| -0.00835 | 0.018193 |
| -0.00846 | 0.018288 |
| -0.00857 | 0.018384 |
| -0.00868 | 0.018481 |
| -0.0088 | 0.018579 |
| -0.00891 | 0.018678 |
| -0.00903 | 0.018777 |
| -0.00915 | 0.018878 |
| -0.00927 | 0.018979 |
| -0.00939 | 0.019082 |
| -0.00951 | 0.019185 |
| -0.00963 | 0.019289 |
| -0.00976 | 0.019395 |
| -0.00989 | 0.019501 |
| -0.01001 | 0.019608 |
| -0.01014 | 0.019716 |
| -0.01028 | 0.019826 |
| -0.01041 | 0.019936 |
| -0.01054 | 0.020048 |
| -0.01068 | 0.02016 |
| -0.01082 | 0.020274 |
| -0.01096 | 0.020389 |
| -0.0111 | 0.020505 |
| -0.01124 | 0.020622 |
| -0.01139 | 0.02074 |
| -0.01154 | 0.020859 |
| -0.01169 | 0.02098 |
| -0.01184 | 0.021102 |
| -0.01199 | 0.021225 |
| -0.01214 | 0.021349 |
| -0.0123 | 0.021475 |
| -0.01246 | 0.021602 |
| -0.01262 | 0.02173 |
| -0.01278 | 0.02186 |
| -0.01295 | 0.021991 |
| -0.01312 | 0.022123 |
| -0.01329 | 0.022257 |

| | |
|---|---|
| -0.01346 | 0.022392 |
| -0.01363 | 0.022529 |
| -0.01381 | 0.022667 |
| -0.01399 | 0.022807 |
| -0.01417 | 0.022948 |
| -0.01436 | 0.023091 |
| -0.01454 | 0.023236 |
| -0.01473 | 0.023382 |
| -0.01492 | 0.023529 |
| -0.01512 | 0.023678 |
| -0.01531 | 0.02383 |
| -0.01551 | 0.023982 |
| -0.01572 | 0.024137 |
| -0.01592 | 0.024293 |
| -0.01613 | 0.024451 |
| -0.01634 | 0.024611 |
| -0.01656 | 0.024773 |
| -0.01678 | 0.024937 |
| -0.017 | 0.025102 |
| -0.01722 | 0.02527 |
| -0.01745 | 0.02544 |
| -0.01768 | 0.025611 |
| -0.01791 | 0.025785 |
| -0.01815 | 0.025961 |
| -0.01839 | 0.026139 |
| -0.01864 | 0.02632 |
| -0.01889 | 0.026502 |
| -0.01914 | 0.026687 |
| -0.0194 | 0.026875 |
| -0.01966 | 0.027064 |
| -0.01992 | 0.027256 |
| -0.02019 | 0.027451 |
| -0.02047 | 0.027648 |
| -0.02074 | 0.027848 |
| -0.02102 | 0.02805 |
| -0.02131 | 0.028255 |
| -0.0216 | 0.028462 |
| -0.0219 | 0.028673 |
| -0.0222 | 0.028886 |
| -0.0225 | 0.029102 |
| -0.02281 | 0.029322 |
| -0.02313 | 0.029544 |
| -0.02345 | 0.029769 |
| -0.02378 | 0.029997 |
| -0.02411 | 0.030229 |
| -0.02444 | 0.030464 |
| -0.02479 | 0.030702 |
| -0.02514 | 0.030944 |
| -0.02549 | 0.031189 |

| | |
|---|---|
| -0.02585 | 0.031438 |
| -0.02622 | 0.03169 |
| -0.02659 | 0.031946 |
| -0.02697 | 0.032206 |
| -0.02736 | 0.03247 |
| -0.02775 | 0.032737 |
| -0.02816 | 0.033009 |
| -0.02856 | 0.033285 |
| -0.02898 | 0.033565 |
| -0.0294 | 0.03385 |
| -0.02983 | 0.034139 |
| -0.03027 | 0.034432 |
| -0.03072 | 0.03473 |
| -0.03118 | 0.035033 |
| -0.03164 | 0.035341 |
| -0.03211 | 0.035654 |
| -0.0326 | 0.035972 |
| -0.03309 | 0.036295 |
| -0.03359 | 0.036623 |
| -0.0341 | 0.036957 |
| -0.03462 | 0.037297 |
| -0.03515 | 0.037642 |
| -0.03569 | 0.037993 |
| -0.03625 | 0.038351 |
| -0.03681 | 0.038714 |
| -0.03739 | 0.039084 |
| -0.03797 | 0.03946 |
| -0.03857 | 0.039843 |
| -0.03919 | 0.040233 |
| -0.03981 | 0.04063 |
| -0.04045 | 0.041035 |
| -0.0411 | 0.041446 |
| -0.04177 | 0.041866 |
| -0.04245 | 0.042293 |
| -0.04314 | 0.042728 |
| -0.04385 | 0.043172 |
| -0.04458 | 0.043624 |
| -0.04532 | 0.044085 |
| -0.04608 | 0.044555 |
| -0.04685 | 0.045034 |
| -0.04765 | 0.045522 |
| -0.04846 | 0.046021 |
| -0.04929 | 0.046529 |
| -0.05014 | 0.047048 |
| -0.05101 | 0.047578 |
| -0.0519 | 0.048119 |
| -0.05281 | 0.048671 |
| -0.05375 | 0.049235 |
| -0.05471 | 0.049811 |

| | |
|---|---|
| -0.05569 | 0.050399 |
| -0.05669 | 0.051 |
| -0.05772 | 0.051614 |
| -0.05878 | 0.052243 |
| -0.05986 | 0.052885 |
| -0.06097 | 0.053542 |
| -0.06211 | 0.054214 |
| -0.06328 | 0.054901 |
| -0.06448 | 0.055605 |
| -0.06572 | 0.056325 |
| -0.06698 | 0.057062 |
| -0.06828 | 0.057818 |
| -0.06962 | 0.058591 |
| -0.07099 | 0.059384 |
| -0.0724 | 0.060197 |
| -0.07386 | 0.06103 |
| -0.07535 | 0.061884 |
| -0.07689 | 0.062761 |
| -0.07847 | 0.06366 |
| -0.08009 | 0.064583 |
| -0.08177 | 0.06553 |
| -0.0835 | 0.066504 |
| -0.08528 | 0.067503 |
| -0.08711 | 0.068531 |
| -0.089 | 0.069587 |
| -0.09095 | 0.070673 |
| -0.09296 | 0.07179 |
| -0.09504 | 0.072939 |
| -0.09718 | 0.074122 |
| -0.0994 | 0.075341 |
| -0.10168 | 0.076596 |
| -0.10405 | 0.077889 |
| -0.1065 | 0.079223 |
| -0.10903 | 0.080598 |
| -0.11164 | 0.082017 |
| -0.11435 | 0.083481 |
| -0.11716 | 0.084993 |
| -0.12007 | 0.086555 |
| -0.12309 | 0.08817 |
| -0.12622 | 0.089839 |
| -0.12946 | 0.091566 |
| -0.13283 | 0.093353 |
| -0.13634 | 0.095204 |
| -0.13997 | 0.097122 |
| -0.14376 | 0.099109 |
| -0.14769 | 0.101171 |
| -0.15179 | 0.10331 |
| -0.15606 | 0.105532 |
| -0.16051 | 0.10784 |

| | |
|---|---|
| -0.16515 | 0.110239 |
| -0.16999 | 0.112735 |
| -0.17504 | 0.115333 |
| -0.18033 | 0.118039 |
| -0.18585 | 0.120859 |
| -0.19163 | 0.123801 |
| -0.19768 | 0.126873 |
| -0.20402 | 0.130081 |
| -0.21068 | 0.133435 |
| -0.21766 | 0.136945 |
| -0.225 | 0.14062 |
| -0.23271 | 0.144472 |
| -0.24083 | 0.148514 |
| -0.24938 | 0.152757 |
| -0.2584 | 0.157218 |
| -0.26792 | 0.161912 |
| -0.27798 | 0.166855 |
| -0.28863 | 0.172069 |
| -0.2999 | 0.177572 |
| -0.31185 | 0.183389 |
| -0.32455 | 0.189544 |
| -0.33804 | 0.196067 |
| -0.35241 | 0.202989 |
| -0.36772 | 0.210343 |
| -0.38407 | 0.218171 |
| -0.40156 | 0.226513 |
| -0.42029 | 0.235421 |
| -0.44039 | 0.244947 |
| -0.462 | 0.255153 |
| -0.48527 | 0.26611 |
| -0.51039 | 0.277896 |
| -0.53756 | 0.290601 |
| -0.56701 | 0.304328 |
| -0.59902 | 0.319195 |
| -0.63389 | 0.335337 |
| -0.67198 | 0.352912 |
| -0.71371 | 0.372103 |
| -0.75958 | 0.393122 |
| -0.81015 | 0.416221 |
| -0.86612 | 0.441695 |
| -0.92829 | 0.469898 |
| -0.99764 | 0.501249 |
| -1.07535 | 0.536258 |
| -1.16285 | 0.575542 |
| -1.26189 | 0.619857 |
| -1.37466 | 0.670136 |
| -1.50387 | 0.72755 |
| -1.65296 | 0.793571 |
| -1.82636 | 0.870086 |

| | |
|---|---|
| -2.02979 | 0.959538 |
| -2.27079 | 1.06514 |
| -2.5595 | 1.19121 |
| -2.90974 | 1.34361 |
| -3.34088 | 1.53055 |
| -3.88066 | 1.76377 |
| -4.57007 | 2.06059 |
| -5.4719 | 2.44749 |
| -6.68638 | 2.96667 |
| -8.38223 | 3.68904 |
| -10.8625 | 4.74177 |
| -14.7199 | 6.37311 |
| -21.2543 | 9.12663 |
| -33.8288 | 14.4061 |
| -63.7422 | 26.9192 |
| -173.437 | 72.6325 |
| -2282.01 | 947.63 |
